# The sinusoidal hematopoietic niche is formed by Jam1a via Notch signaling in the zebrafish kidney

**DOI:** 10.1016/j.isci.2023.106508

**Published:** 2023-03-27

**Authors:** Mao Kondo, Koki Kimura, Jingjing Kobayashi-Sun, Shiori Yamamori, Makoto Taniguchi, David Traver, Isao Kobayashi

**Affiliations:** 1 Division of Life Sciences, Graduate School of Natural Science and Technology, Kanazawa University, Kanazawa, Ishikawa 920-1192, Japan; 2 Department of Clinical Engineering, Faculty of Health Sciences, Komatsu University, Komatsu, Ishikawa 923-0961, Japan; 3 Faculty of Biological Science and Technology, Institute of Science and Engineering, Kanazawa University, Kanazawa, Ishikawa 920-1192, Japan; 4 Department of Life Science, Medical Research Institute, Kanazawa Medical University, Uchinada, Ishikawa 920-0293, Japan; 5 Department of Cellular and Molecular Medicine, University of California at San Diego, La Jolla, CA 92093, USA

**Keywords:** Biological sciences, Physiology, Animal physiology, Molecular physiology

## Abstract

The zebrafish is a unique model to understand hematopoietic niches as hematopoietic stem/progenitor cells are maintained in the kidney. However, little is known about which cell types in the kidney play a role in hematopoietic niches. Here, we demonstrate that the sinusoidal endothelium is an essential and conserved niche component in the zebrafish kidney. Histological analysis revealed that *runx1:mCherry*^+^ hematopoietic cells were predominantly detected in the dorsolateral region of the kidney where sinusoids are highly developed. Loss of Junctional adhesion molecule 1a (Jam1a), which is expressed in both sinusoidal endothelial cells and hematopoietic cells, resulted in a remarkable reduction in sinusoids and a defect in hematopoietic niches. We found that Jam1a regulates *jagged-1a* expression in vascular endothelial cells to form a sinusoidal structure in the kidney. Collectively, these data suggest that sinusoids are formed by Jam1a via endothelial Notch signaling to provide hematopoietic niches in the zebrafish kidney.

## Highlights

• Sinusoids are predominantly formed in the dorsolateral region of the kidney
• Hematopoietic cells are localized along the sinusoidal endothelium in the kidney
• Jam1a regulates *jagged-1a* expression to form the sinusoidal hematopoietic niche

## Graphical abstract

**Figure:**
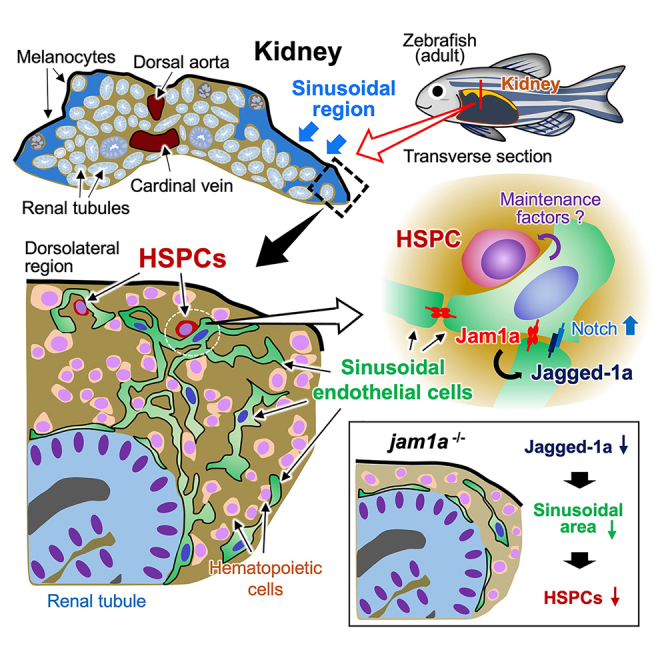

## Introduction

Hematopoietic stem cells (HSCs) are rare cells with the remarkable ability to both self-renew and generate all mature blood cell types over the life span of an individual. HSCs present within the adult bone marrow or newborn cord blood are by far the most widely utilized human stem cells in the clinic. To date, however, *ex vivo* expansion and maintenance of HSCs has not been possible for regenerative medicine approaches. A major obstacle has been the lack of understanding of the key cell types and signaling pathways that comprise the HSC niche, which is defined as the cellular and molecular components that regulate HSC quiescence, self-renewal, and differentiation.^1^^,^^2^^,^^3^ Studies in the murine bone marrow have identified some essential cellular components of the HSC niche, such as osteoblasts,^4^^,^^5^^,^^6^ sinusoidal endothelial cells,^7^^,^^8^^,^^9^^,^^10^ perivascular cells,^9^^,^^11^^,^^12^^,^^13^ mesenchymal stem cells,^14^ macrophages,^15^ and megakaryocytes.^16^^,^^17^^,^^18^ These niche cells highly express supporting factors for HSCs, also termed “niche factors”, including angiopoietin 1,^6^ stem cell factor (Scf),^9^^,^^19^ C-X-C motif ligand 12 (Cxcl12; also known as stromal cell-derived factor 1 [SDF1]),^11^^,^^20^ Jagged-1,^10^ and thrombopoietin.^21^^,^^22^ Nevertheless, there still remains controversy about the precise HSC niche, and a better understanding of the elements that regulate HSCs is required.

The zebrafish is an excellent model for a parallel view of the HSC niche. Many valuable tools and experimental methods have been established for the study of HSCs in zebrafish (e.g., transgenic/mutant animals, genome editing, transplantation assays, cell culture assays, etc.). Despite the high conservation of hematopoiesis at the cellular and molecular levels, the nature of the niche appears at face value to be different as the adult hematopoietic organ in teleost fish is the kidney. Various stages of hematopoietic cells and mature blood cells are observed in interstitial tissue of the kidney, termed the “kidney marrow”.^23^^,^^24^^,^^25^ Although, the zebrafish kidney is thus of great importance for understanding hematopoietic niches, little is known regarding which cell types play a role in hematopoietic niches in the kidney. This is due, at least in part, to the lack of specific HSC markers in zebrafish. Recently, we established a method to purify zebrafish HSCs from the adult kidney using a double-transgenic line, *gata2a:GFP*; *runx1:mCherry*. Based on these transgenes, three distinct hematopoietic cell populations can be resolved in kidney marrow cells (KMCs), *gata2a:GFP*^+^ *runx1:mCherry*^+^ (hereafter denoted as *gata2a*^+^ *runx1*^+^), *gata2a*^−^ *runx1*^+^, and *gata2a*^+^ *runx1*^−^. Transcriptome analysis revealed that *gata2a*^+^ *runx1*^+^ cells displayed typical molecular hallmarks of HSCs, whereas *gata2a*^−^ *runx1*^+^ cells and *gata2a*^+^ *runx1*^−^ cells showed expression signatures of erythroid/myeloid cells and lymphoid cells, respectively. Competitive transplantation assays demonstrated that 100 *gata2a*^+^ *runx1*^+^ cells fully reconstituted hematopoiesis over 16 weeks in irradiated zebrafish, indicating that zebrafish HSCs are highly enriched in the *gata2a*^+^ *runx1*^+^ fraction in the kidney.^26^^,^^27^

Here, we demonstrate that the sinusoidal endothelium is essential for hematopoietic niches in the zebrafish kidney. Histological analysis revealed that *runx1:mCherry*^+^ hematopoietic cells were predominantly observed in the dorsolateral (DL) region of the kidney, where sinusoidal capillaries were abundant. Loss of junctional adhesion molecule 1a (Jam1a, also known as F11r), which is expressed by both sinusoidal endothelial cells and hematopoietic cells, resulted in a remarkable reduction in sinusoids and a defect in hematopoietic niches. We uncovered that Jam1a regulates *jagged-1a* expression in vascular endothelial cells to form a sinusoidal structure that provides hematopoietic niches in the kidney. Our data provide evidence that the sinusoidal endothelium is an evolutionarily conserved component of hematopoietic niches in vertebrates.

## Results

### Hematopoietic cells are localized along the sinusoidal endothelium in the dorsolateral region of the kidney

**Figure 1:**
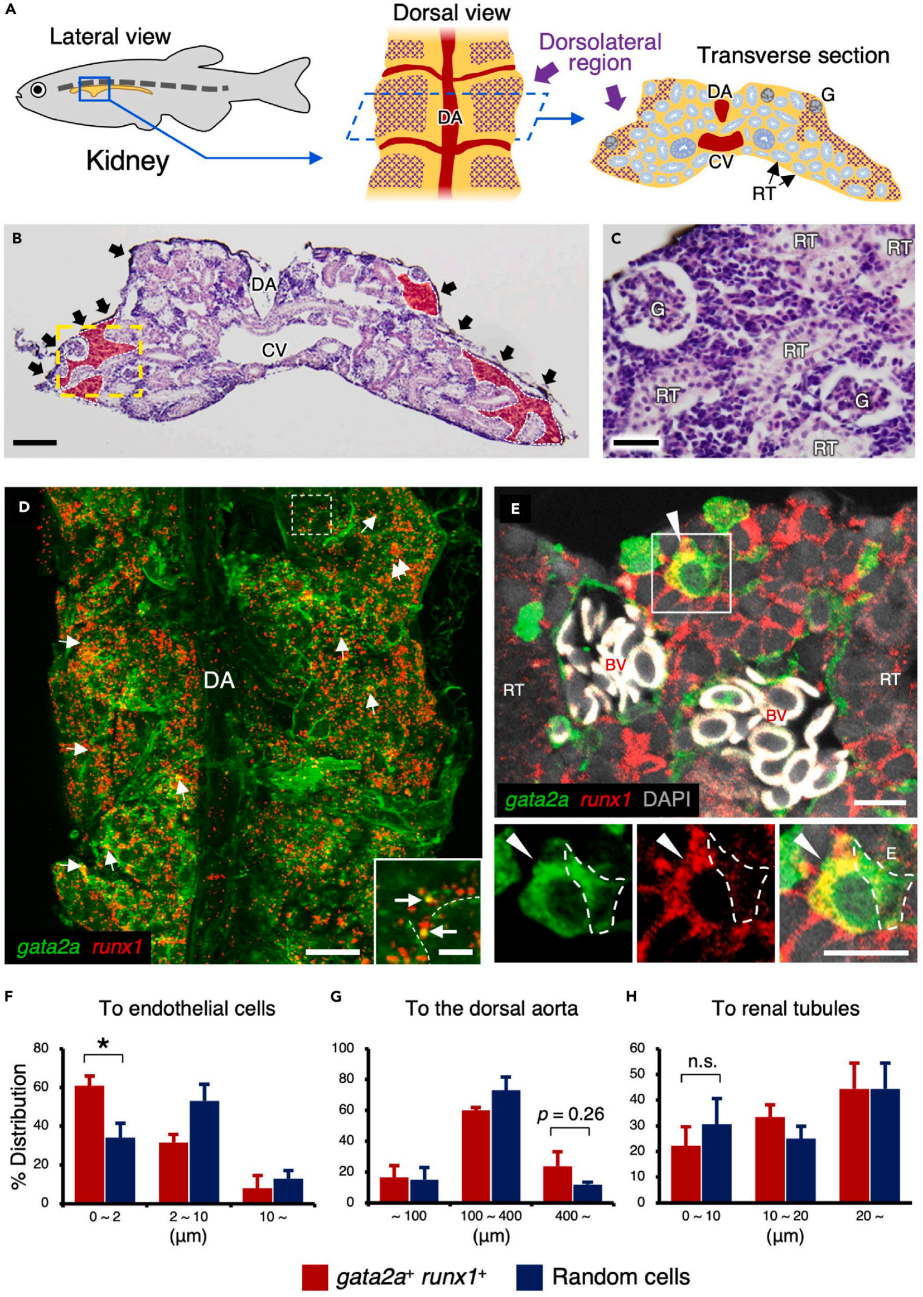
*gata2a*^+^*runx1*^+^ HSPCs are closely associated with vascular endothelial cells in the kidney (A) Schematic diagram of zebrafish kidney. Lateral view (left), dorsal view (middle), and transverse section of the kidney (right) are shown. The purple cross-hatching portion denotes the dorsolateral region of the kidney. (B and C) Hematoxylin and eosin staining of zebrafish kidney. Red areas and black arrows denote kidney marrow and melanocytes observed in the dorsal surface of the kidney. A high magnification view of the yellow dotted area is shown in C. (D) Dorsal view of a *gata2a:GFP*; *runx1:mCherry* kidney. Inset shows the high magnification view of the white dotted area. Arrows and dotted lines indicate *gata2a*^+^*runx1*^+^ cells and endothelial cells, respectively. (E) Transverse section of a *gata2a:GFP*; *runx1:mCherry* kidney. Insets show the green (left), red (middle), and merged channel (right) of the white boxed area. Arrowheads indicate a *gata2a*^+^*runx1*^+^ cell, and white dotted lines outline a *gata2a:GFP*^+^ vascular endothelial cell. The section is oriented dorsal side up. Erythrocytes within blood vessels are observed in white due to auto-fluorescence. (F–H) Distance (μm) of individual *gata2a*^+^*runx1*^+^ cells or randomly selected cells from the endothelium, dorsal aorta, or renal tubule (total 82 cells from 3 zebrafish for the endothelium and dorsal aorta and total 36 cells from 3 zebrafish for renal tubules; error bars, s.d.). ∗p < 0.05; n.s., no significance; DA, dorsal aorta; CV, cardinal vein; G, glomerulus; RT, renal tubule; BV, blood vessels. Bars, 100 μm (B); 40 μm (C); 200 μm (D); 20 μm (insets in D); 10 μm (E).

We first examined the structure of the zebrafish kidney by histological analysis. Zebrafish kidneys flank the dorsal aorta and cardinal vein and contain multiple types of renal epithelia including glomeruli, proximal and distal tubules, and collecting ducts. Although, there was no clear separation in renal structures as seen in mammalian kidneys (e.g., cortex and medulla), glomeruli tended to be distributed along the dorsal to lateral surface. We found that KMCs were predominantly observed in the DL region of the kidney (Figures 1A–1C). It has recently been reported that melanocytes dorsally cover hematopoietic organs in aquatic animals, termed the “melanocyte umbrella”, which is suggested to protect hematopoietic stem/progenitor cells (HSPCs) from UV light.^28^ Consistent with this observation, zebrafish kidneys were dorsally covered by melanocytes (Figure 1B), suggesting that the kidney marrow is formed mainly under the melanocyte umbrella. To investigate the distribution of HSPCs, we performed whole-mount immunohistochemistry in kidneys dissected from *gata2a:GFP*; *runx1:mCherry* animals. Expression of *runx1:mCherry* was restricted in hematopoietic cells, whereas *gata2a:GFP* was detected not only in hematopoietic cells but also in vascular endothelial cells. Although the majority of *runx1:mCherry*^+^ hematopoietic cells did not express *gata2a:GFP*, a few *gata2a*^+^ *runx1*^+^ cells were sporadically detected in the DL region of the kidney (Figure 1D). We found that *gata2a*^+^ *runx1*^+^ cells were closely associated with *gata2a:GFP*^+^ vascular endothelial cells. A transverse section of the kidney also showed that *gata2a*^+^ *runx1*^+^ cells were in contact with a *gata2a:GFP*^+^ vascular endothelial cell that typically shows spindle to long thin shapes with weak GFP expression and is morphologically distinguishable from *gata2a:GFP*^+^ hematopoietic cells (Figure 1E). By measuring the distance of individual *gata2a*^+^ *runx1*^+^ cells or randomly selected cells to the *gata2a:GFP*^+^ endothelium, we found that approximately 60% of *gata2a*^+^ *runx1*^+^ cells were within 2 μm of the endothelium, which was in significantly higher frequency than randomly selected cells (Figure 1F). While not significant, *gata2a*^+^ *runx1*^+^ cells also tended to be detected at far distances from the dorsal aorta (Figure 1G). However, there was no difference in the distance to renal tubules (Figure 1H). Taken together, these data suggest that HSPCs closely interact with vascular endothelial cells in the kidney.

**Figure 2:**
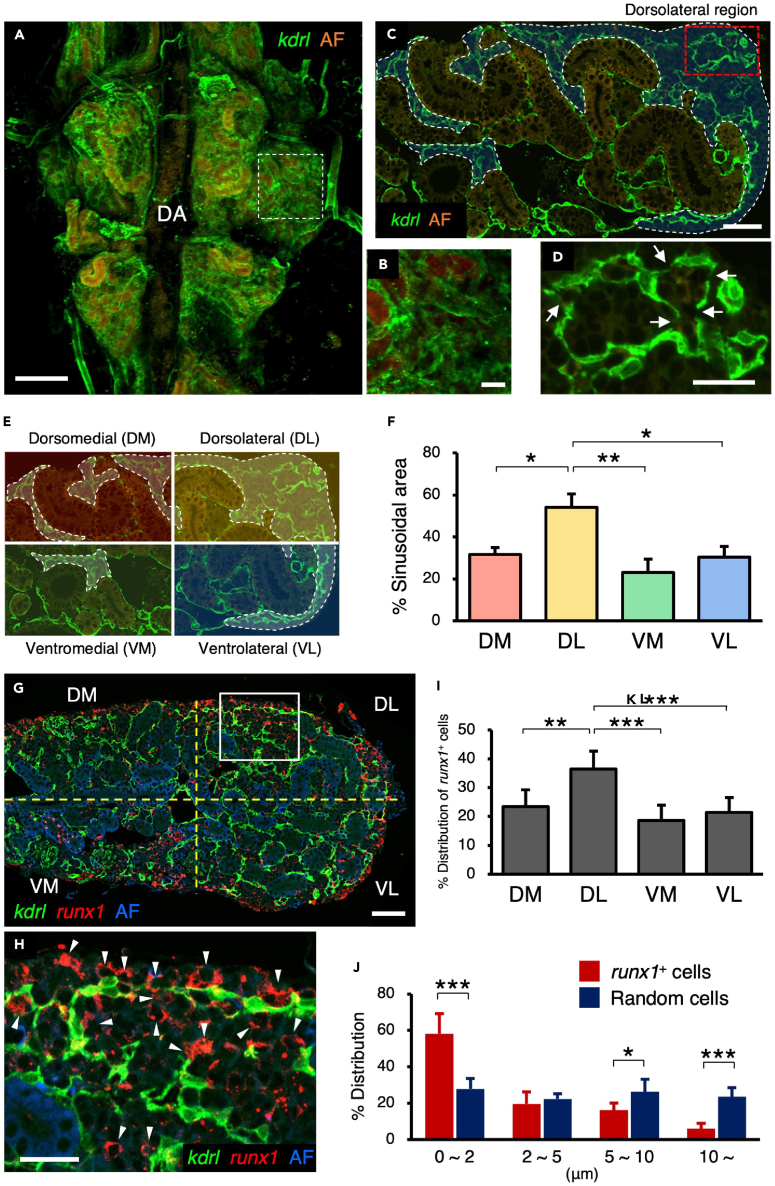
Sinusoids are formed in the dorsolateral region of the kidney (A and B) Dorsal view of a *kdrl:Cerulean* kidney. A high magnification view of the white dotted area is shown in B. (C and D) Transverse section of a *kdrl:Cerulean* kidney. Blue areas outlined by the white dotted line denote the sinusoidal area. A high magnification view of the red dotted area is shown in D. Arrows indicate the fenestra in the sinusoidal endothelium. (E and F) Quantification of sinusoidal area in the kidney. Kidney tissue was subdivided into 4 regions, dorsomedial (DM), dorsolateral (DL), ventromedial (VM), and ventrolateral (VL), and the percentage of sinusoidal area was quantified in each region (mean ± s.e.m; n = 6). (G and H) Transverse section of a *kdrl:Cerulean*; *runx1:mCherry* kidney. A high magnification view of the white boxed area is shown in H. Arrowheads in H indicate *runx1*^+^ cells detected in the sinusoidal area. AF in A–C, G, and H denotes auto-fluorescence of renal tubules or erythrocytes. (I and J) Percent distribution of *runx1*^+^ cells in each region (I) and distance (μm) of individual *runx1*^+^ cells or randomly selected cells from the *kdrl:Cerulean*^+^ sinusoidal endothelium (J) (total 904 *runx1*^+^ cells and 183 random cells from 4 zebrafish; error bars, s.d.). ∗p < 0.05; ∗∗p < 0.01; DA, dorsal aorta; Bars, 200 μm (A); 40 μm (B, C, G); 20 μm (D, H).

To further investigate vasculature in the kidney, we utilized *kdrl:Cerulean* animals, which express Cerulean under the control of vascular specific *kdrl* enhancers.^29^ Whole-mount and section immunohistochemistry revealed that there were at least three types of vascular endothelium in the kidney, the endothelium lining along the major blood vessel (e.g., dorsal aorta, cardinal vein, and their branches); the endothelium surrounding the renal tubule (“renal endothelium”); and the sinusoidal endothelium (Figure S1A). The renal endothelium, which comprises small blood vessels to carry away the recovered solute, was entirely observed in the kidney. In contrast, the sinusoidal endothelium, which forms fenestrated capillaries, was predominantly observed in the DL region (Figures 2A–2D and S1A). Kidney tissue was subdivided into 4 regions, dorsomedial (DM), DL, ventromedial (VM), and ventrolateral (VL), and sinusoidal area was quantified in each region. The percentage of sinusoidal area in the DL region was approximately 54.0%, which was markedly higher than the DM (31.7%), VM (23.0%), or VL region (30.3%) (Figures 2E and 2F). The distribution of sinusoids appeared to be correlated with the distribution of melanocytes as both were mainly localized in the dorsal side of the kidney (Figures S1B and S1C). To investigate the co-localization of hematopoietic cells and sinusoidal endothelium in the kidney, the *kdrl:Cerulean* line was combined with the *runx1:mCherry* line and kidney tissue was histologically analyzed. There were large numbers of *runx1*^+^ hematopoietic cells distributed around the *kdrl*^+^ sinusoids in the kidney (Figures 2G and 2H). The percentage of *runx1*^+^ cells distributed within the DL region was approximately 36.5%, significantly higher than that in the other regions (Figure 2I). Moreover, the percentage of *runx1*^+^ cells distributed within 2 μm of the sinusoidal endothelium was approximately 58.2%, whereas the percentage of those distributed over 10 μm was only 6.1% (Figure 2J). These data suggest that sinusoids are predominantly formed in the DL region of the kidney, and most hematopoietic cells are distributed adjacent to the sinusoidal endothelium. Both glomeruli and blood vessels were found to be more abundant dorsally in the kidney, especially in the DL region where they tended to be distributed within the region of the sinusoids; however, no localization of *runx1*^+^ cells around glomeruli or blood vessels was observed (Figures S2A–S2D).

### Loss of Jam1a results in a reduction of HSPCs in the kidney

In mammals, Jam1 (also known as Jam-A) is expressed in hematopoietic cells, vascular endothelial cells, and renal epithelium, and is involved in cell-cell interactions based on homophilic and/or heterophilic binding with other Jam molecules.^30^^,^^31^^,^^32^ We examined Jam1a expression in the adult kidney under a *kdrl:Cerulean* background by immunohistochemistry and found that Jam1a was detected in both hematopoietic cells and sinusoidal endothelium in the kidney marrow (Figures S3A and S3B).

To examine the role of Jam1a in adult hematopoiesis, we generated a genetic mutant line, *jam1a*^*sd43*^, which is predicted to contain a premature stop codon due to a 10 base-pair deletion in exon 3 (Figures S3C and S3D). Similar to Jam1 knockout mice,^33^^,^^34^ homozygous *jam1a*^*sd43*^ zebrafish are partially viable into adulthood and fertile, and there were no obvious morphological abnormalities, whereas *jam1a*^*sd43*^ adult males showed slightly lower body weight than age-matched wild-type males (Figures S3E–S3G). Western blotting analysis revealed that the wild-type form of the Jam1a protein was completely lost in *jam1a*^*sd43*^ animals (Figures S3H and S3I). Jam1a was strongly expressed in the epithelium of collecting ducts and tubules in the kidney, as has been shown in the human kidney,^31^ whereas this expression was never detected in *jam1a*^*sd43*^ animals (Figures S3J and S3K). Despite the strong expression of Jam1a in the collecting ducts and tubules, there were no obvious morphological abnormalities in the renal tissue, suggesting that Jam1a may be dispensable for renal functions. It has previously been shown in the zebrafish embryo that loss of Jam1a results in a reduction of developing HSPCs in the dorsal aorta due to low levels of Notch signaling.^35^ Consistent with this observation, approximately 87% of *jam1a*^*sd43*^ embryos showed reduced to no expression of the HSPC marker gene, *runx1*, in the dorsal aorta (Figures S3L–S3N).

To test if *jam1a*^*sd43*^ animals display a hematopoietic defect in the adult kidney, the *jam1a*^*sd43*^ line was combined with a double-transgenic line, *kdrl:Cre*; *bactin2:loxP-STOP-loxP-DsRed* (hereafter referred to as *kdrl-sw*), which labels nearly all adult blood cells with dsRed except for mature erythrocytes.^26^^,^^36^ Flow cytometric (FCM) analysis revealed that the absolute numbers of *kdrl-sw*^–^ erythrocytes, *kdrl-sw*^+^ non-erythrocyte blood cells, and granulocytes were approximately 41%, 35%, and 55% lower in *jam1a*^*sd43*^ animals than those in age-matched wild-type animals, respectively, whereas the numbers of lymphoid and precursor cells were unchanged (Figures S4A–S4D).

**Figure 3:**
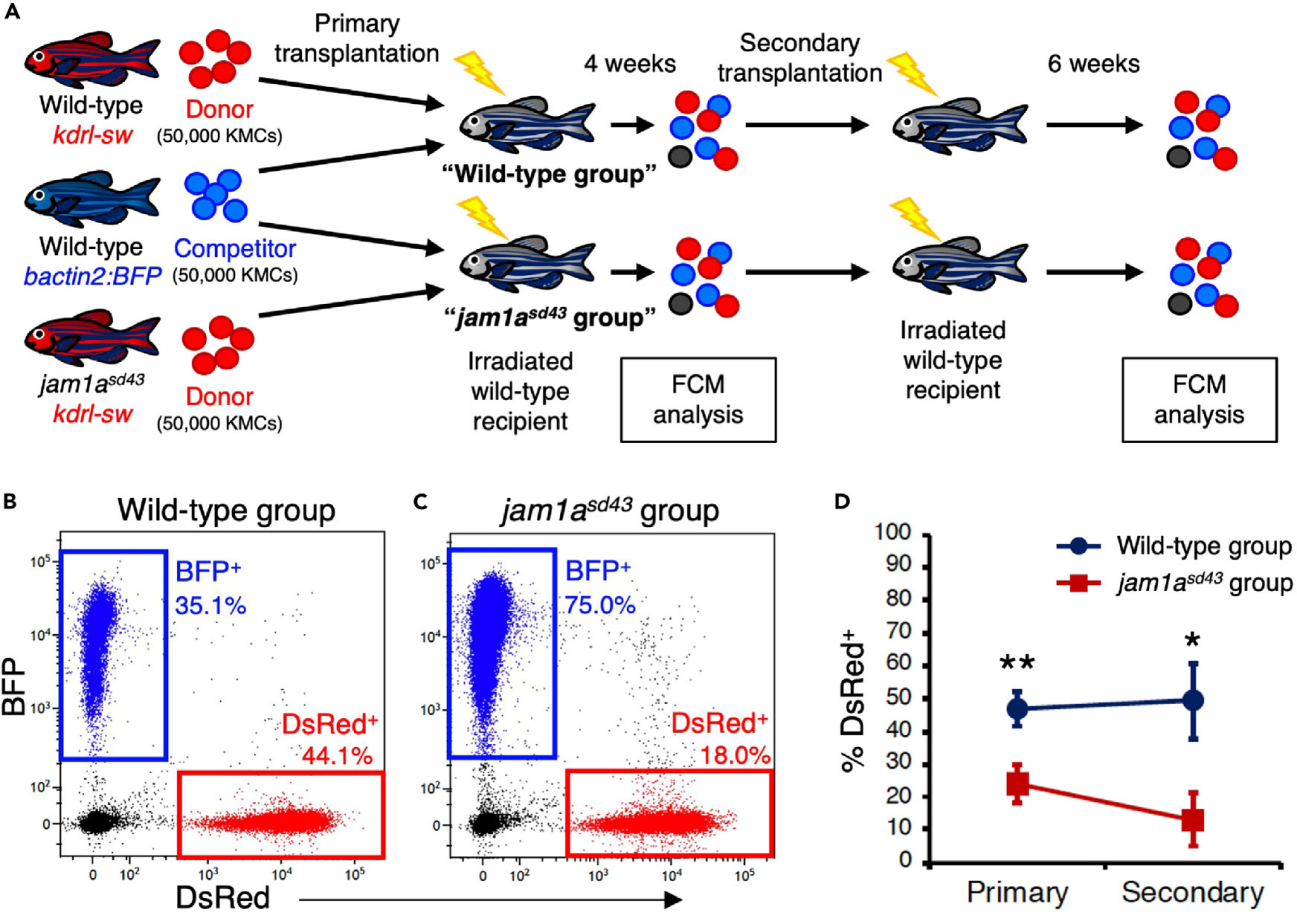
Serial transplantation assay for wild-type and *jam1a*^*sd4*^ KMCs (A) Experimental procedure for serial transplantation assay. DsRed-labeled KMCs from wild-type or *jam1a*^*sd43*^ animals were co-transplanted with equivalent numbers of BFP-labeled wild-type KMCs into sub lethally irradiated wild-type recipients (wild-type or *jam1a*^*sd43*^ group). At 4 weeks post-transplantation (wpt), KMCs from each recipient group were analyzed by FCM and further transplanted into secondary recipients, followed by FCM analysis at 6 wpt. (B and C) Representative result of FCM analysis in recipients of wild-type or *jam1a*^*sd43*^ group. (D) Percentages of dsRed^+^ cells within the total BFP^+^ or dsRed^+^ cells in each group (mean ± s.e.m; n = 35 (wild-type) or 33 (*jam1a*^*sd43*^) for primary; n = 14 (wild-type) or 16 (*jam1a*^*sd43*^) for secondary). ∗p < 0.05; ∗∗p < 0.01.

The potential for hematopoietic reconstitution can be evaluated through an *in vivo* competitive repopulation assay, in which the contributions of donor- and competitor-derived cells are compared in irradiated recipients.^26^^,^^37^ DsRed-labeled wild-type or *jam1a*^*sd43*^ kMCs (donors; *kdrl-sw* background) were co-transplanted with equal numbers of blue fluorescent protein (BFP)-labeled wild-type KMCs (competitors; *bactin2:BFP* background) into sub lethally irradiated recipients (wild-type or *jam1a*^*sd43*^ group). At 4 weeks post-transplantation (wpt), KMCs from each recipient group were further transplanted into secondary recipients, followed by analysis of KMCs at 6 wpt (Figure 3A). In the wild-type group, the percentage of donor-derived dsRed^+^ cells within the total dsRed^+^ and BFP^+^ cells was closed to 50% in both primary and secondary transplantations, confirming the reliability and validity of this transplantation assay. In contrast, contribution was approximately 24.1% in primary recipients and 13.2% in secondary recipients in the *jam1a*^*sd43*^ group (Figures 3B–3D), indicating that mutation of *jam1a* results in low levels of hematopoietic reconstitution activity. We also evaluate the homing capacity of *jam1a*^*sd43*^ kMCs. DsRed-labeled wild-type or *jam1a*^*sd43*^ kMCs were co-injected with equal numbers of BFP-labeled wild-type KMCs into wild-type recipients (wild-type or *jam1a*^*sd43*^ group), followed by analysis of KMCs at 2 days post-transplantation (dpt). The percentage of donor-derived dsRed^+^ cells within the total dsRed^+^ and BFP^+^ cells in the wild-type group was approximately 42.3%, whereas that in the *jam1a*^*sd43*^ group was 11.7% (Figures S5A–S5C). These data suggest that mutation of *jam1a* also affects the homing capacity of KMCs.

**Figure 4:**
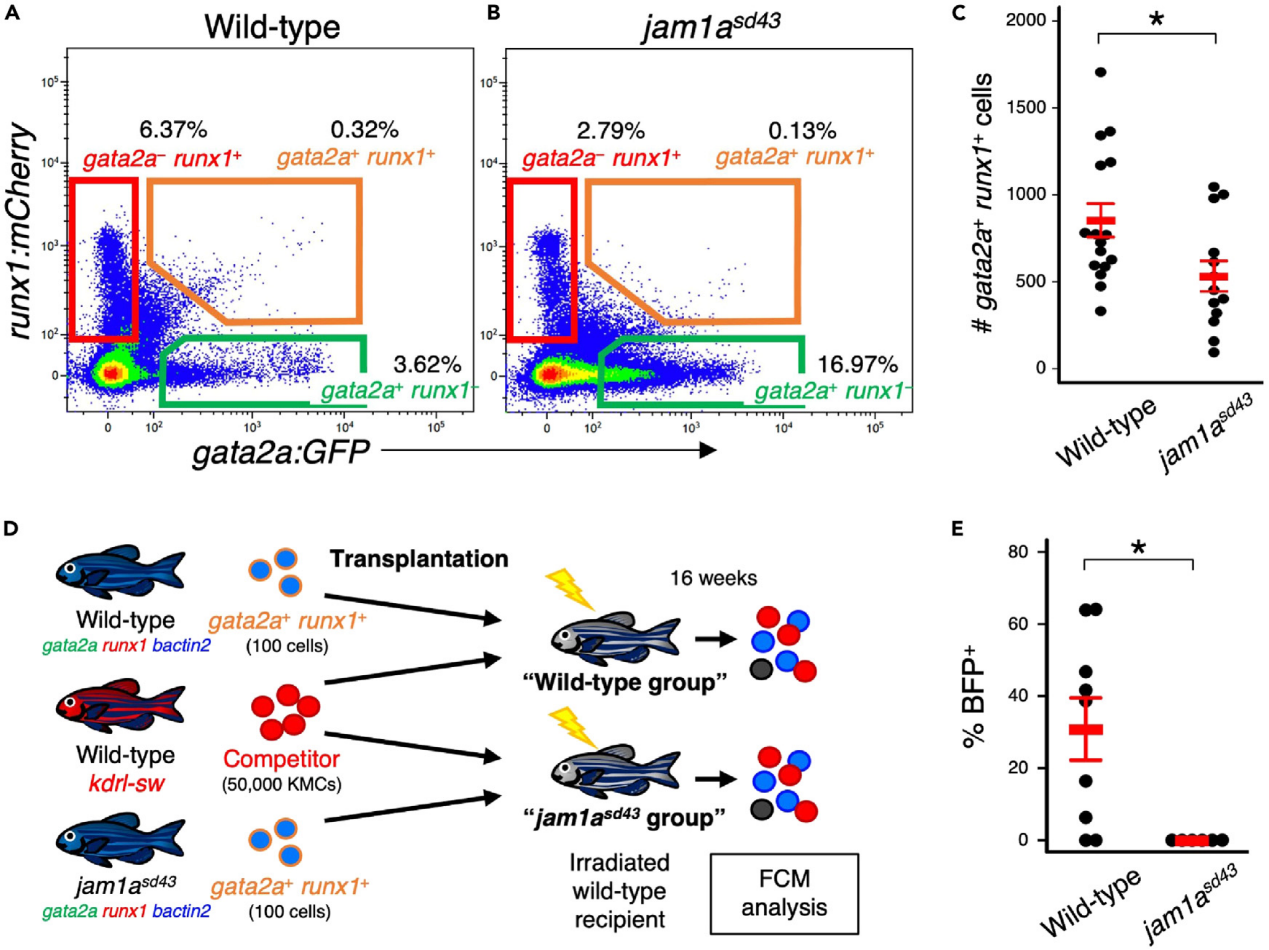
Loss of Jam1a reduces long-term hematopoietic reconstitution activity (A and B) Representative result of FCM analysis in KMCs from wild-type or *jam1a*^*sd43*^ animals under a *gata2a:GFP*; *runx1:mCherry* background. (C) Absolute numbers of *gata2a*^+^*runx1*^+^ cells in the wild-type or *jam1a*^*sd43*^ kidney (mean ± s.e.m; n = 16 [wild-type] or 13 [*jam1a*^*sd43*^]). (D) Experimental procedure for competitive repopulation assay using *gata2a*^+^*runx1*^+^ cells. (E) Percentages of BFP^+^ cells within total BFP^+^ or dsRed^+^ cells in each group (mean ± s.e.m; n = 9 [wild-type] or 6 [*jam1a*^*sd43*^]). ∗p < 0.05.

To compare the number of HSPCs between wild-type and *jam1a*^*sd43*^ animals, we performed FCM analysis in KMCs under *gata2a:GFP*; *runx1:mCherry* background (Figures S6A and S6B). The absolute numbers of *gata2a*^+^ *runx1*^+^ cells were approximately 38% lower in *jam1a*^*sd43*^ animals than wild-type animals (Figures 4A–4C), while there was no significant difference in the percentage of *gata2a*^+^ *runx1*^+^ cells in KMCs (Figure S7A). We also observed that both the percentage and absolute number of *gata2a*^−^ *runx1*^+^ cells (erythroid and myeloid cells) were approximately 30% lower in *jam1a*^*sd43*^ animals than those in wild-type animals, whereas those of *gata2a*^+^ *runx1*^−^ cells (lymphoid cells) were approximately 50% higher (Figures S7B and S7C).

In order to evaluate the hematopoietic reconstitution activity of *gata2a*^+^ *runx1*^+^ cells in *jam1a*^*sd43*^ animals, wild-type, and *jam1a*^*sd43*^ animals under the background of *gata2a:GFP*; *runx1:mCherry*; *bactin2:BFP* were used for competitive repopulation assay. One hundred *gata2a*^+^ *runx1*^+^ cells from wild-type or *jam1a*^*sd43*^ animals were co-transplanted with DsRed-labeled wild-type KMCs (competitors) into sub lethally irradiated recipients (wild-type or *jam1a*^*sd43*^ group) (Figure 4D). The percentage of donor-derived BFP^+^ cells in the wild-type group was approximately 30.8% at 16 wpt, whereas that in the *jam1a*^*sd43*^ group was only 0.02% (Figure 4E). These data suggest that mutation of *jam1a* affects the long-term hematopoietic reconstitution of HSCs.

The differentiation potential of *gata2a*^+^ *runx1*^+^ cells was evaluated by an *in vitro* colony assay in the presence of Epoa or Csf3b (Gcsfb). We did not observe significant differences between wild-type and *jam1a*^*sd43*^ cells in the percentage of colony-forming units (CFU) in either condition (Figures S7D and S7E). Because there was a reduction in the number of erythrocytes and granulocytes in the *jam1a*^*sd43*^ kidney despite the unchanged differentiation potential of *gata2a*^+^ *runx1*^+^ cells, the expression levels of *epoa* and *csf3b* were compared between the wild-type and *jam1a*^*sd43*^ kidney by quantitative polymerase chain reaction (qPCR). We observed a large reduction in the expression of both genes in the *jam1a*^*sd34*^ kidney (Figure S7F), suggesting that decreased erythrocytes and neutrophils are attributed to decreased stimulating factors for erythropoiesis and granulopoiesis in the *jam1a*^*sd34*^ kidney.

### Loss of Jam1a causes a defect in hematopoietic niches in the kidney

**Figure 5:**
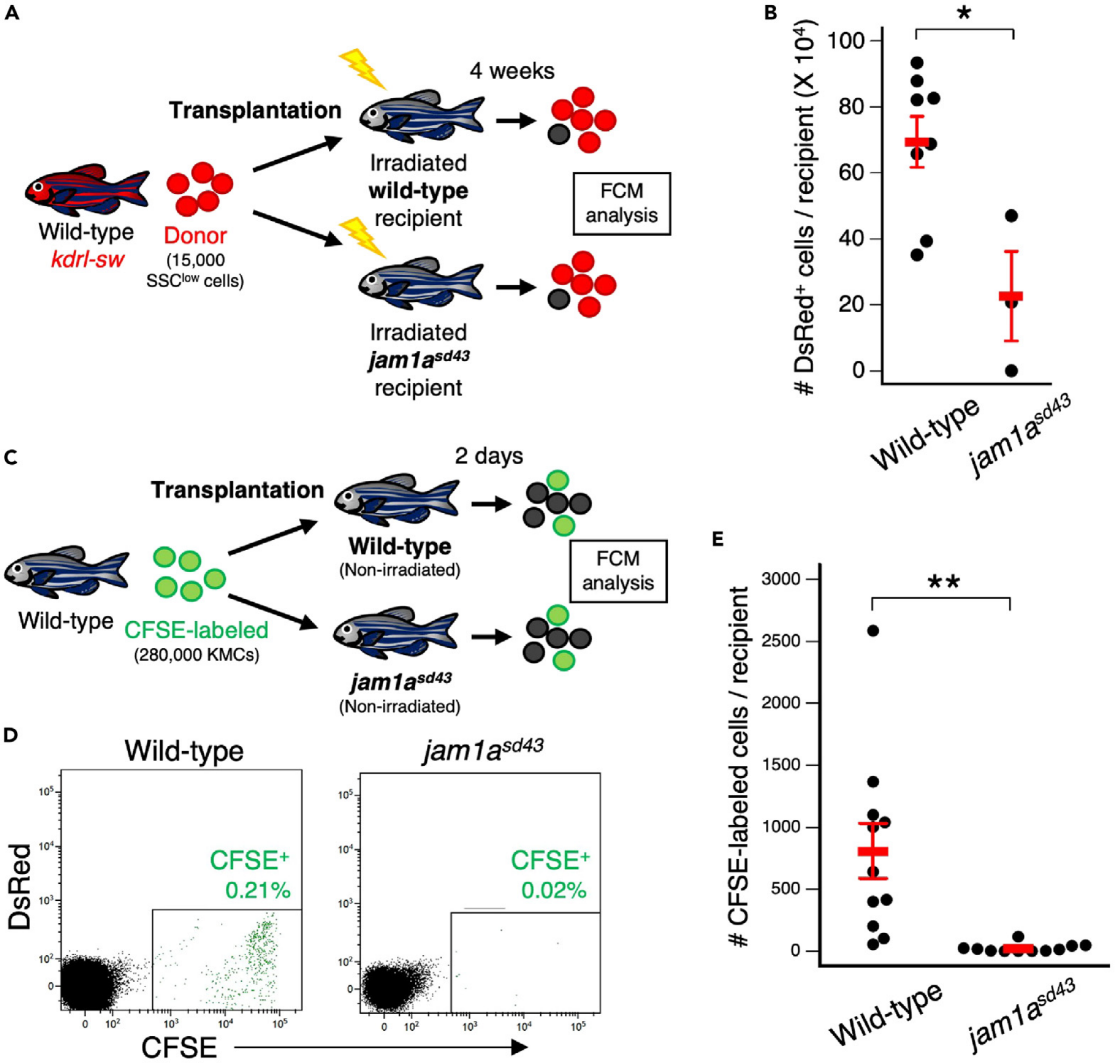
Loss of Jam1a results in a defect in hematopoietic niches (A) Experimental procedure for transplantation assay using wild-type or *jam1a*^*sd43*^ recipients. (B) Absolute numbers of dsRed^+^ cells in wild-type or *jam1a*^*sd43*^ recipients (mean ± s.e.m; n = 8 [wild-type] or 3 [*jam1a*^*sd43*^]). (C) Experimental procedure for homing assay using wild-type or *jam1a*^*sd43*^ recipients. (D) Representative result of FCM analysis in wild-type or *jam1a*^*sd43*^ recipients. (E) Absolute numbers of CFSE-labeled cells in wild-type or *jam1a*^*sd43*^ recipients (mean ± s.e.m; n = 11 for each). ∗p < 0.05; ∗∗p < 0.01.

Since Jam1a was expressed not only in hematopoietic cells but also in sinusoidal endothelial cells in the kidney, we next evaluated the capacity of hematopoietic niches in *jam1a*^*sd43*^ animals by transplantation assays. Side scatter^low^ (SSC^low^) cells (containing HSPCs) were collected from wild-type *kdrl-sw* animals and transplanted into irradiated wild-type or *jam1a*^*sd43*^ recipients, followed by FCM analysis at 4 wpt (Figure 5A). The absolute numbers of donor-derived dsRed^+^ cells were approximately 3-fold lower in *jam1a*^*sd43*^ recipients than in wild-type recipients (Figure 5B), suggesting that mutation of *jam1a* causes a defect in hematopoietic niches. We also investigated if wild-type hematopoietic cells can be home to the kidney of *jam1a*^*sd43*^ animals. CFSE-labeled wild-type KMCs were transferred to non-irradiated wild-type or *jam1a*^*sd43*^ recipients (280,000 cells/recipient), and KMCs in each recipient at 2 dpt were analyzed by FCM (Figure 5C). The absolute number of CFSE-labeled KMCs in wild-type recipients was 810 ± 222 (n = 11, ± s.e.m.), whereas that in *jam1a*^*sd43*^ recipients was only 24 ± 11 (n = 11, ± s.e.m.) (Figures 5D and 5E).

**Figure 6:**
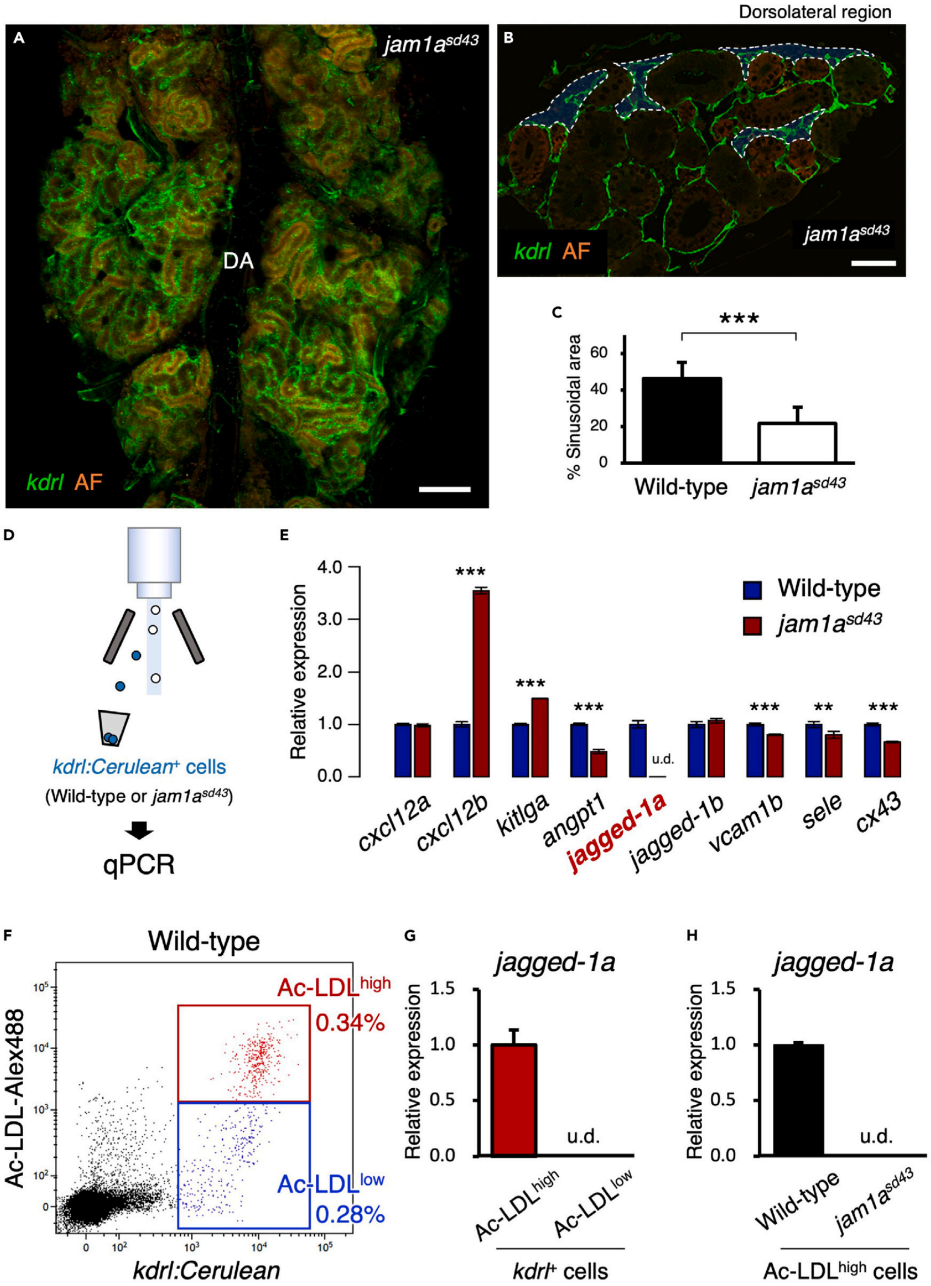
Sinusoids are reduced in the *jam1a*^*sd43*^ kidney (A) Dorsal view of a *jam1a*^*sd43*^*kdrl:Cerulean* kidney. DA, dorsal aorta. (B) Transverse section of a *jam1a*^*sd43*^*kdrl:Cerulean* kidney. Blue areas outlined by the white dotted line denote the sinusoidal area. AF in A and B denotes auto-fluorescence of renal tubules. (C) Percentages of sinusoidal area in the wild-type or *jam1a*^*sd43*^ kidney. Error bars, s.d. (n = 6 for each). (D) Schematic diagram of qPCR analysis in vascular endothelial cells from wild-type or *jam1a*^*sd43*^ kidneys. (E) Expression of niche factor genes in vascular endothelial cells from wild-type or *jam1a*^*sd43*^ kidneys. Error bars, s.d. (n = 4 for each). (F) Representative result of FCM analysis in wild-type *kdrl:Cerulean* animals injected with Alexa Fluor 488-conjugated Ac-LDL. *kdrl:Cerulean*^+^ endothelial cells were subdivided into two populations, Ac-LDL^high^ and Ac-LDL^low^. (G and H) Expression of *jagged-1a* in Ac-LDL^high^ or Ac-LDL^low^ endothelial cells of wild-type animals (G) or in Ac-LDL^high^ endothelial cells from wild-type or *jam1a*^*sd43*^ kidneys (H). Error bars, s.d. (n = 4 for each). u.d., undetected. Bars, 200 μm (A); 40 μm (B); ∗∗p < 0.01; ∗∗∗p < 0.001.

Given the defect in hematopoietic niches in *jam1a*^*sd43*^ animals, we next investigated the vasculature in the *jam1a*^*sd43*^ kidney in a *kdrl:Cerulean* background. Although the renal endothelium was normally observed, the sinusoidal endothelium was remarkably reduced in *jam1a*^*sd43*^ animals compared to wild-type animals (Figure 6A). The percentage of sinusoidal area in the wild-type kidney was approximately 46.2%, whereas the *jam1a*^*sd43*^ kidney contained only 21.7% (Figures 6B and 6C).

In mammals, Cxcl12 is known to be expressed in stromal cells and endothelial cells in the bone marrow to regulate HSC homing.^11^^,^^20^ Given the defect in hematopoietic cell homing to the *jam1a*^*sd43*^ kidney, we next compared *cxcl12* expression between wild-type and *jam1a*^*sd43*^ kidneys. Whole-mount fluorescent *in situ* hybridization analysis revealed that the expression of *cxcl12a* was predominantly detected in the renal endothelium, and there were no obvious differences between wild-type and *jam1a*^*sd43*^ kidneys in terms of expression patterns and levels (Figures S8A and S8B). qPCR analysis also revealed that the expression of *cxcl12a* and *cxcl12b* was unchanged between the wild-type and *jam1a*^*sd43*^ kidney (Figure S8C).

To investigate the molecular mechanisms underlying the defect in hematopoietic niches in the *jam1a*^*sd43*^ kidney, we performed expression analysis of some niche factor genes in *kdrl:Cerulean*^+^ endothelial cells from wild-type or *jam1a*^*sd43*^ kidneys. Although expression of *cxcl12b* and *kitlga* (*kit ligand a*) was upregulated, *angpt1* (*angiopoietin 1*), *jagged-1a* (*jagged canonical Notch ligand 1a*), *vcam1b* (*vascular cell adhesion molecule 1b*), *sele* (*selectin E*), and *cx43* (*connexin 43*) were downregulated in *jam1a*^*sd43*^ endothelial cells compared to wild-type endothelial cells, notably, *jagged-1a* was nearly undetected in *jam1a*^*sd43*^ endothelial cells (Figures 6D and 6E). While sinusoids and other blood vessels share most vascular markers, sinusoidal endothelial cells endocytose acetylated low-density lipoprotein (Ac-LDL).^38^^,^^39^ In order to compare *jagged-1a* expression between sinusoids and other endothelial cells, Alexa Fluor 488-conjugated Ac-LDL was intravenously injected into *kdrl:Cerulean* animals, and kidney cells were analyzed by FCM. In the wild-type kidney, *kdrl:Cerulean*^+^ endothelial cells were subdivided into two populations based on uptake of Ac-LDL, Ac-LDL^high^, and Ac-LDL^low^. Expression of *jagged-1a* was only detected in Ac-LDL^high^ *kdrl:Cerulean*^+^ endothelial cells (Figures 6F and 6G), suggesting that *jagged-1a* is predominantly expressed in sinusoidal endothelial cells in the kidney. Although the percentage of Ac-LDL^high^ within the *kdrl:Cerulean*^+^ population was unchanged in the *jam1a*^*sd43*^ kidney (Figures S8D–S8F), expression of *jagged-1a* was undetected in Ac-LDL^high^ *kdrl:Cerulean*^+^ endothelial cells in *jam1a*^*sd43*^ animals (Figure 6H).

### Jagged-1a is required for sinusoid formation in the kidney

**Figure 7:**
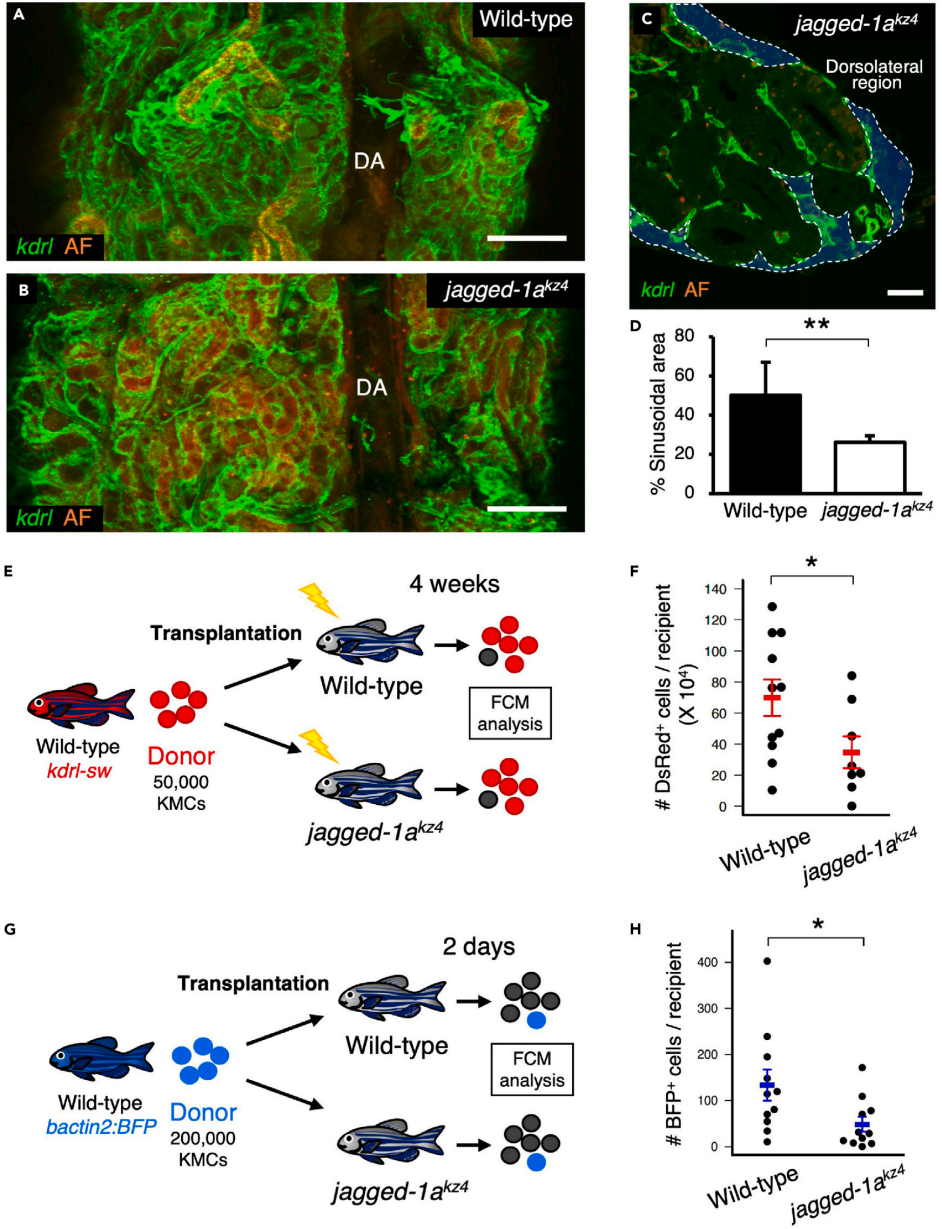
Loss of Jagged-1a results in a defect in hematopoietic niches (A and B) Dorsal view of a wild-type or *jagged-1a*^*kz4*^ kidney. (C) Transverse section of a *jagged-1a*^*kz4*^ kidney under a *kdrl:Cerulean* background. Blue areas outlined by the white dotted line denote the sinusoidal area. (D) Percentages of sinusoidal area in the wild-type or *jagged-1a*^*kz4*^ kidney. Error bars, s.d. (n = 4 for each). (E) Experimental procedure for transplantation assay using wild-type or *jagged-1a*^*kz4*^ recipients. (F) Absolute numbers of dsRed^+^ cells in wild-type or *jagged-1a*^*kz4*^ recipients (mean ± s.e.m; n = 11 [wild-type] or 8 [*jagged-1a*^*kz4*^]). (G) Experimental procedure for homing assay using wild-type or *jagged-1a*^*kz4*^ recipients. (H) Absolute numbers of BFP^+^ cells in wild-type or *jagged-1a*^*kz4*^ recipients (mean ± s.e.m; n = 11 for each). AF in A-C denotes auto-fluorescence of renal tubules. ∗p < 0.05; ∗∗p < 0.01. Bars, 200 μm (A and B); 20 μm (C).

In mice, Jagged-1 is known to be a proangiogenic regulator and is required for promoting HSC self-renewal in the bone marrow.^10^^,^^40^ To investigate the role of Jagged-1a in hematopoiesis in the zebrafish kidney, we generated *jagged-1a* mutant zebrafish (*jagged-1a*^*kz4*^) by the CRISPR/Cas9 system. qPCR analysis confirmed that the wild-type form of *jagged-1a* mRNA was lost in the *jagged-1a*^*kz4*^ kidney (Figures S9A and S9B). We examined the vasculature of the *jagged-1a*^*kz4*^ kidney in a *kdrl:Cerulean* background. Similar to *jam1a*^*sd43*^ kidneys, *jagged-1a*^*kz4*^ kidneys also displayed a reduction in sinusoidal endothelium, whereas renal endothelium was unaffected (Figures 7A and 7B). Section immunocytochemistry revealed that the sinusoidal area in the *jagged-1a*^*kz4*^ kidney was approximately 48% lower than the wild-type kidney (Figures 7C and 7D). To examine the capacity of hematopoietic niches in the *jagged-1a*^*kz4*^ kidney, DsRed-labeled wild-type KMCs were transplanted into irradiated wild-type or *jagged-1a*^*kz4*^ recipients, followed by FCM analysis at 4 wpt. We found that the absolute numbers of donor-derived dsRed^+^ cells were approximately 50% lower in *jagged-1a*^*kz4*^ recipients than in wild-type recipients (Figures 7E and 7F). In addition, homing assays also revealed that the absolute number of BFP^+^ cells that home to the kidney was reduced in *jagged-1a*^*kz4*^ recipients compared to wild-type recipients (Figures 7G and 7H), suggesting that mutation of *jagged-1a* phenocopies a defect in hematopoietic niches in *jam1a*^*sd43*^ animals.

**Figure 8:**
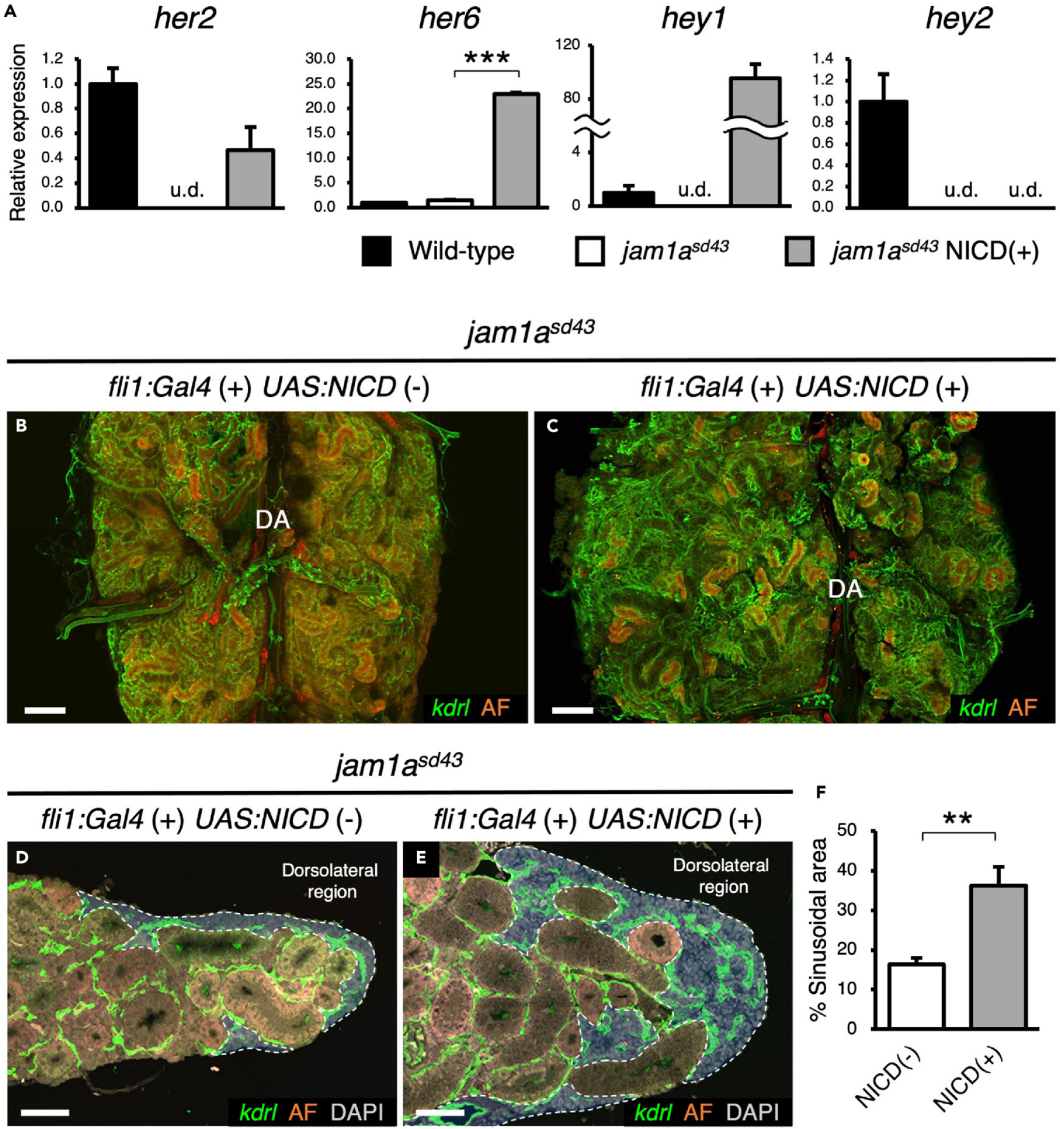
Forced activation of Notch recovers sinusoids in the *jam1a*^*sd43*^ kidney (A) Relative expression of Notch target genes, *her2*, *her6*, *hey1*, and *hey2*, in *kdrl:Celurean*^+^ Ac-LDL^high^ sinusoidal endothelial cells from wild-type, *jam1a*^*sd43*^, or *jam1a*^*sd43*^ expressing NICD. u.d., undetected. Error bars, s.d. (n = 3 for each). (B–E) Dorsal view (B and C) and transverse section (D and E) of a *jam1a*^*sd43*^ kidney expressing or not NICD under control of *fli1:Gal4* induction. Blue areas outlined by the white dotted line in D and E denote the sinusoidal area. AF denotes auto-fluorescence of renal tubules. (F) Percentages of sinusoidal area in the *jam1a*^*sd43*^ kidney expressing or not NICD under control of *fli1:Gal4* induction. Error bars, s.d. (n = 4 for each). ∗∗p < 0.01; ∗∗∗p < 0.001. Bars, 200 μm (B and C); 40 μm (D and E).

Since Jagged-1a is a Notch ligand that activates Notch signaling via interaction with Notch receptors, we next investigated whether enforced activation of Notch in vascular endothelial cells can recover sinusoids in the *jam1a*^*sd43*^ kidney. A dominant activator of Notch signaling, the Notch intracellular domain (NICD), was expressed in vascular endothelial cells in *jam1a*^*sd43*^ animals using the *fli1:Gal4*; *UAS:NICD* line.^35^ We first examined the expression of Notch target genes, *her2* (also known as *hes5*), *her6* (also known as *hes1*), *hey1*, and *hey2*, in *kdrl:Cerulean*^+^ Ac-LDL^high^ sinusoidal endothelial cells in wild-type, *jam1a*^*sd43*^, and *jam1a*^*sd43*^ with forced expression of NICD by qPCR. Compared to wild-type sinusoidal endothelial cells, *her2*, *hey1*, and *hey2* were downregulated in *jam1a*^*sd43*^ sinusoidal endothelial cells, whereas *her6* was almost unchanged. In contrast, forced expression of NICD in *jam1a*^*sd43*^ sinusoidal endothelial cells resulted in upregulation of *her2*, *her6*, and *hey1* (Figure 8A). Histological analysis revealed that the sinusoidal structure was remarkably increased in the kidney of NICD (+) *jam1a*^*sd43*^ animals (Figures 8B–8F), suggesting that formation of the sinusoidal structure is required for Jagged-1a-derived Notch signaling in endothelial cells. However, transplantation of wild-type BFP^+^ cells into NICD (−) or NICD (+) *jam1a*^*sd43*^ recipients showed that the absolute number of donor-derived BFP^+^ cells was unchanged between these two types of *jam1a*^*sd43*^ recipients (Figures S10A and S10B). This is likely because HSPCs cannot home to the kidney without Jam1a expression in vascular endothelial cells. Indeed, homing assays revealed that forced expression of NICD in vascular endothelial cells did not increase the number of wild-type BFP^+^ cells homing to the *jam1a*^*sd4*^ kidney (Figures S10C and S10D). Taken together, these data suggest that Jam1a regulates sinusoidal niche formation in the kidney by regulating *jagged-1a* expression in vascular endothelial cells, and Jam1a also plays a role in recruiting hematopoietic cells to the kidney.

## Discussion

In the present study, we demonstrate that sinusoidal endothelium is a key component of the HSC niche in the zebrafish kidney, and that Jam1a plays a pivotal role in its formation and function in large part via regulation of *jagged-1a* expression in vascular endothelial cells.

Endothelial cells that comprise sinusoidal blood vessels have been implicated directly in regulating HSCs in murine bone marrow. Most CD150^+^ CD48^−^ CD41^−^ Lineage^−^ HSCs are in contact with sinusoidal endothelial cells,^7^ and endothelial-specific deletion of Jagged-1 results in depletion of the HSC pool in the bone marrow.^10^ In addition, conditional deletion of Scf, which regulates HSC proliferation, in endothelial cells also leads to HSC depletion in the bone marrow.^9^ *In vivo* imaging analysis in the bone marrow revealed that sinusoids are a major target for HSC homing and engraftment.^8^ Consistent with these observations, we showed in zebrafish that *runx1*^+^ hematopoietic cells predominantly localized along the sinusoidal endothelium in the DL region of the kidney. The efficiency of HSPC engraftment was lower in *jam1a*^*sd43*^ or *jagged-1a*^*kz4*^ recipients, whereas the sinusoidal structure was recovered in *jam1a*^*sd43*^ animals by forced Notch activation in vascular endothelial cells. These data suggest that the major role of Jagged-1a in HSC niches is to generate sinusoidal niches and that direct interactions with sinusoids may be essential for HSC maintenance and self-renewal in the zebrafish kidney.

Sinusoids are commonly observed in both adult and embryonic hematopoietic organs in mammals, including the bone marrow,^7^^,^^8^^,^^9^^,^^10^ spleen,^41^ and fetal liver.^42^^,^^43^ In zebrafish embryos, HSCs arise from the dorsal aorta and move to the transient hematopoietic organ, the caudal hematopoietic tissue (CHT), an equivalent organ to the mammalian fetal liver. Previous studies have shown that sinusoidal structures are highly developed in the CHT in the zebrafish embryo.^44^^,^^45^ Live-imaging analysis of HSPCs in CHT revealed that HSPCs are enwrapped in sinusoidal endothelial cells, which are described as “endothelial cuddling”. This specific interaction with sinusoidal endothelial cells results in close contact between HSCs and a single mesenchymal stromal cell, leading to oriented cell division of the HSC.^46^ These observations, in combination with our data, suggest that sinusoids are both an evolutionarily and developmentally conserved component of HSC niches.

The expression of *Jam1* in HSCs is highly conserved in human, mouse, and zebrafish^30^^,^^47^; however, there have been no reports of its function in the hematopoietic niche. We demonstrated that the number of cells homing to the kidney was decreased in either case when *jam1a*^*sd43*^ animals were used as donor or recipient, suggesting that Jam1a regulates hematopoietic cell homing to the kidney in both a cell autonomous and non-cell autonomous manner. Since Jam1a expression in hematopoietic niches is required for hematopoietic cell homing, forced expression of NICD in endothelial cells is not sufficient to rescue niche functions in the *jam1a*^*sd43*^ kidney, but NICD expression is sufficient to restore sinusoidal structures. Collectively, these data suggest that Jam1a not only regulates *jagged-1a* expression in vascular endothelial cells to form a sinusoidal structure, but also functions to recruit hematopoietic cells to the kidney. Given that Jam1 expression in hematopoietic cells and vascular endothelial cells is highly conserved, the function of Jam1 in hematopoiesis may be conserved among vertebrates.

In zebrafish, melanocytes that located in the dorsal region of the kidney have been shown to play a role in protecting HSPCs from UV light-induced DNA damage. The melanocyte umbrella of the hematopoietic organ is highly evolutionarily conserved in aquatic animals, including jawless fish, teleost fish, and amphibians.^28^ We found that *runx1*^+^ hematopoietic cells were predominantly detected in the DL region of the kidney that is covered by melanocytes. Moreover, the distribution of sinusoids in the kidney was correlated with that of melanocytes, supporting the idea that hematopoietic niches are protected by melanocytes in aquatic animals.

Since teleost fish and mammals share a common ancestor, the zebrafish kidney provides a complementary system to understand the universal regulatory mechanisms of vertebrate HSCs. Further studies in zebrafish and other vertebrates will define core cellular components that have been conserved over evolutionary time, and lead to new discoveries regarding the molecular cues required for HSC maintenance and expansion *ex vivo*.

### Limitations of the study

In this study, we found in zebrafish that sinusoids are predominantly formed in the DL region of the kidney, and most *runx1*^+^ hematopoietic cells are distributed adjacent to the sinusoidal endothelium; however, there are several limitations. First, we cannot show if *gata2a*^+^ *runx1*^+^ HSPCs are closely associated with sinusoidal endothelial cells due to the technical difficulty of visualizing *kdrl*^+^ sinusoidal endothelial cells under the background of *gata2a:GFP* in the kidney. Ac-LDL cannot also be used to visualize sinusoidal endothelial cells as Alexa 488, the fluorescent label used for Ac-LDL, fades upon removal of melanin from the kidney by hydrogen peroxide treatment. Since much of the sinusoidal endothelium overlaps with the distribution of the melanin pigment, the sinusoidal endothelium in the kidney cannot be visualized without the removal of melanin. Second, it is not possible to show if *gata2a*^+^ *runx1*^+^ HSPCs home to the DL region of the kidney. Given that only a few *gata2a*^+^ *runx1*^+^ HSPCs are expected to home to the kidney by transplantation of KMCs, no reliable data on HSPC homing can be presented in zebrafish. Third, although we showed that Jam1a regulates the formation of a sinusoidal structure via *jagged-1a* expression in vascular endothelial cells, the regulatory mechanisms of *jagged-1a* expression by Jam1a require further exploration.

## STAR★Methods

### Key resources table

**Table:** 

REAGENT or RESOURCE	SOURCE	IDENTIFIER
**Antibodies**		

Chicken anti-GFP	Aves	Cat#GFP-1020; RRID:AB_10000240
Rabbit polyclonal anti-RFP	Abcam	Cat#ab34771; RRID:AB_777699
Goat Anti-Chicken IgY H&L (Alexa Fluor 488)	Abcam	Cat#ab150173; RRID:AB_2827653
Donkey Anti-Rabbit IgG H&L (Alexa Fluor 647)	Abcam	Cat#ab150075; RRID:AB_2752244
Goat Anti-Mouse IgG H&L (Alexa Fluor 647)	Abcam	Cat#ab150115; RRID:AB_2687948
Mouse anti-Jam1a (2O7)	This paper	N/A
Anti-Digoxigenin-AP, Fab fragments	Sigma	Cat#1093274; RRID:AB_514497
Anti-Digoxigenin-POD, Fab fragments	Sigma	Cat#11207733910; RRID:AB_514500
Mouse anti-GAPDH	Abcam	Cat#ab8245; RRID:AB_2107448
Goat anti-mouse IgG HRP-conjugated	Jackson ImmunoResearch	Cat#115-035-166; RRID:AB_10015289

**Chemicals, peptides, and recombinant proteins**		

Low Density Lipoprotein From Human Plasma, Acetylated, Alexa Fluor 488 Conjugate	Thermo Fisher Scientific	Cat#L23380
Liberase TM Research Grade	Roche	Cat#5401119001
SYTOX Red Dead Cell Stain	Thermo Fisher Scientific	Cat#S34859
5(6)-Carboxyfluorescein diacetate N-succinimidyl ester	Sigma	Cat#21888
Polyinosinic acid potassium salt	Sigma	Cat#P4154
SuperScript III Reverse Transcriptase	Thermo Fisher Scientific	Cat#18080093
Exonuclease I	Takara	Cat#2650A
Terminal Transferase(Tdt), recombinant	Sigam	Cat#3333566001
MightyAmp DNA Polymerase Ver.2	Thermo Fisher Scientific	Cat#R071A
TB Green Premix Ex Taq II (Tli RNaseH Plus)	Takara	Cat#RR820S
Erythropoietin a (zebrafish)	Kobayashi et al.^26^	N/A
Colony stimulating factor 3b (zebrafish)	Kobayashi et al.^26^	N/A
DIG RNA Labeling Mix	Roche	Cat#1277073
T7 RNA Polymerase	Roche	Cat#10881767001
TWEEN 20	Sigma	Cat#P9416
Proteinase K, recombinant, PCR Grade	Roche	Cat#3115887001
Nitroblue tetrazolium chloride (NBT)	Roche	Cat#11383213001
5-Bromo-4-chloro-3-indolyl-phosphate, 4-toluidine salt (BCIP)	Roche	Cat#11383221001
Blocking Reagent	Roche	Cat#11096176001
Mayer's Hematoxylin Solution	Wako	Cat#131-09665
1% Eosin Y Solution	Wako	Cat#051-06515
Agarose, low gelling temperature	Sigma	Cat#A9414

**Critical commercial assays**		

MEGAshortscript T7 Transcription Kit	Thermo Fisher Scientific	Cat#AM1354
mirVana miRNA Isolation Kit, with phenol	Thermo Fisher Scientific	Cat#AM1560
mMESSAGE mMACHINE SP6 Transcription Kit	Thermo Fisher Scientific	Cat#AM1340
RNeasy Mini Kit	QIAGEN	Cat##74104
ReverTra Ace qPCR RT Master Mix with gDNA Remover	Toyobo	Cat#FSQ-301
QIAquick PCR Purification Kit	QIAGEN	Cat#28104
TSA Plus Cyanine 3 System	Perkin Elmer	Cat#NEL744001KT
NuPAGE 4-12% Bis-Tris Gel	Novax	Cat#NP0335BOX
Immobilon-P Membrane	Millipore	Cat#IPVH00010
SuperSignal West Pico Chemiluminescent Substrate	Thermo Fisher Scientific	Cat#34077

**Experimental models: Organisms/strains**		

Zebrafish AB∗ wild type	Zebrafish International Resource Center	ZDB-GENO-960809-7
*jam1a*^*sd43*^	This paper	N/A
*jagged-1a*^*kz4*^	This paper	N/A
*Tg(gata2a:GFP)*^*la3*^	Balla et al.^48^	N/A
*Tg(Mmu.Runx1:NLS-mCherry)*^*cz2010*^	Tamplin et al.^46^	N/A
*Tg(bactin2:loxP-BFP-loxP-DsRed)*^*sd27*^	Kobayashi et al.^35^	N/A
*Tg(kdrl:Cre)*^*s898*^	Bertrand et al.^36^	N/A
*Tg(bactin2:loxP-STOP-loxP-DsRed)*^*sd5*^	Bertrand et al.^36^	N/A
*Tg(kdrl:NTR-cerulean)*^*sd24*^	Page et al.^29^	N/A
*Tg(fli1:Gal4)*^*ubs4*^	Zygmunt et al.^49^	N/A
*Tg(UAS:NICD)*^*kca3*^	Burns et al.^50^	N/A

**Oligonucleotides**		

Primers	This paper	See Table S1

**Software and algorithms**		

Kaluza software, ver1.3	Beckman Coulter	N/A
TCapture software, ver 4.3.0.602	Tucsen Photonics	N/A
Fluoview FV10i-SW software, ver. 2.1.1	Olympus	N/A

### Resource availability

#### Lead contact

Further information and requests for resources and reagents should be directed to and will be fulfilled by the lead contact, Isao Kobayashi (ikobayashi@se.kanazawa-u.ac.jp).

#### Materials availability

This study did not generate new unique reagents.

### Experimental model and subject details

#### Zebrafish husbandry

Zebrafish strains, AB∗, *jam1a*^*sd43*^, *jagged-1a*^*kz4*^, *Tg(gata2a:GFP)*^*la3*^ (ref.^48^^,^^51^), *Tg(Mmu.Runx1:NLS-mCherry)*^*cz2010*^ (here denoted as *runx1:mCherry*),^46^ *Tg(bactin2:loxP-BFP-loxP-DsRed)*^*sd27*^ (here denoted as *bactin2:BFP*),^35^ *Tg(kdrl:Cre)*^*s898*^ (ref.^36^), *Tg(bactin2:loxP-STOP-loxP-DsRed)*^*sd5*^ (ref.^36^), and *Tg(kdrl:NTR-cerulean)*^*sd24*^ (ref.^29^), *Tg(fli1:Gal4)*^*ubs4*^ (ref.^49^), *Tg(UAS:NICD)*^*kca3*^ (ref.^50^), were raised in a circulating aquarium system (AQUA) at 28.5°C in a 14/10 h light/dark cycle and maintained in accordance with guidelines of the Committee on Animal Experimentation of Kanazawa University. Age-matched adult wild-type and *jam1a*^*sd43*^ animals (3 to 6-month-old males/females) were used for all experiments. Sex was not considered as a variable in this study.

### Method details

#### Generation of mutant zebrafish

For CRISPR/Cas9-mediated generation of a mutant line, the guide RNA (gRNA) was designed to target exon 3 of *jam1a* or exon2 of *jagged-1a* and was synthesized as previously described.^52^^,^^53^ Two complementary oligonucleotides corresponding to the target sequence were annealed and ligated into the *pT7-gRNA* vector. The gRNA was then synthesized using a linearized vector with MEGAshortscript T7 Transcription Kit (Thermo Fisher Scientific) and purified by mirVana miRNA Isolation Kit (Thermo Fisher Scientific). *Cas9* mRNA was synthesized using a linearized *pCS2-Cas9* vector with mMESSAGE mMACHINE SP6 Transcription Kit (Thermo Fisher Scientific) and purified by RNeasy Mini Kit (Qiagen). gRNA (50 pg/nL) and *Cas9* mRNA (150 pg/nL) were co-injected into one-cell stage embryos. Fish from the F1 generation were screened by genomic PCR. For *jam1a*^*sd43*^, the mutant allele was identified by sequencing. For *jagged-1a*^*kz4*^, due to a large deletion in the *jagged-1a* genomic loci, the mutant allele was determined by genomic PCR and was confirmed by RT-PCR. The sequences of the designed gRNA and primers used for genotyping were listed in Table S1.

#### Administration

Alexa Fluor 488-conjugated (AF488) acetylated low-density lipoprotein (Ac-LDL) (Thermo Fisher Scientific) was diluted at the final concentration of 66.7 ng/μL in phosphate buffered saline (PBS). Adult zebrafish were retro-orbitally injected with 7 μL of AF488-Ac-LDL solution. At 3 h post-injection, kidneys were dissected to prepare cells for flow cytometric (FCM) analysis.

#### Cell preparation and flow cytometry

Kidney marrow cells (KMCs) were prepared as previously described.^26^ Cells were obtained by pipetting of a dissected kidney in 1 mL of ice-cold 2% fetal bovine serum (FBS) in PBS (2% FBS/PBS). After centrifugation, the pellet was gently mixed with 1 mL of distilled water by pipetting to lyse erythrocytes by osmotic shock. Subsequently, 1 mL of 2X PBS was added. For preparation of vascular endothelial cells, a dissected kidney was digested with Liberase TM (Roche) in PBS for 1 h at 37°C. Cells were then filtered through a 40-stainless mesh and washed with 2% FBS/PBS by centrifugation. Just before FCM analysis, the Sytox Red (Thermo Fisher Scientific) was added to exclude dead cells. FCM acquisition and cell sorting were performed on a FACS Canto 2 (BD Biosciences) or FACS Aria III (BD Biosciences). Data analysis was performed using the Kaluza software (ver. 1.3, Beckman Coulter). The absolute number of cells was calculated by flow cytometry based on the acquisition events, maximum acquisition times, and the percentage of each cell fraction.

For homing assays, KMCs were stained with 5 μM of 5(6)-Carboxyfluorescein diacetate N-succinimidyl ester (CFSE) (Sigma) in PBS for 10 min at 25°C. Cells were then washed twice with 2% FBS/PBS by centrifugation.

#### qPCR

For sorted cells, whole-transcript amplification and double-strand cDNA synthesis was performed as previously described.^26^ Cells were directly sorted in a lysis buffer containing 1 μg/mL of polyinosinic-polycytidylic acid, and total RNA was extracted using RNeasy Mini Kit. Reverse transcription (RT) was performed using Super Script III (Thermo Fisher Scientific) and an RT primer, which contains oligo-dT, T7 promoter, and PCR target region sequences. After digestion of remaining RT primers by exonuclease I (Takara), a poly-A tail was added to the 3’ ends of the first-strand cDNAs using terminal transferase (Sigma). The second-strand DNA was then synthesized using MightyAmp DNA polymerase (Thermo Fisher Scientific) and a tagging primer, which contains oligo-dT and PCR target region sequences. PCR amplification was performed using a suppression primer, which allow to amplify small-size DNA that contains complementary sequences at both ends of the template DNA. The amplified double-strand cDNA was purified using QIAquick PCR Purification Kit (Qiagen). For the kidney tissue, total RNA was extracted using RNeasy Mini Kit (Qiagen), and cDNA was synthesized using ReverTra Ace qPCR RT Master Mix (Toyobo). Quantitative real-time PCR (qPCR) assays were performed using TB Green Premix Ex Taq II (TaKaRa) on a ViiA 7 Real-Time PCR System according to manufacturer’s instructions (Thermo Fisher Scientific). Primers used for qPCR were listed in Table S1.

#### X-ray irradiation and transplantation

Three to six zebrafish were placed in a petri dish containing with the system water, and animals were then semi-lethally irradiated with X-ray on a Faxitron RX-650 (Faxitron, 130 kVp, 1.15 Gy/min) for 20 min or MX-80Labo (Medixtec, 40 kVp, 0.6 Gy/min) for 2X 19 min (approximately 23 Gy). After 2 days post-irradiation, animals were transferred with cells using a retro-orbital injection method.^54^

#### Colony assay

A recombinant protein of zebrafish erythropoietin a (Epoa) and colony stimulating factor 3b (Csf3b) (also known as granulocyte colony stimulating factor b (Gcsfb)) was generated as previously described.^26^ For a colony assay, *gata2a*^+^ *runx1*^+^ cells were sorted into a round-bottom 96-well plate filled with the 1X ERDF condition medium containing 20% FBS, 2.5% carp serum, and 600 ng/mL of Epo or Csf3b. Cells were cultured at 30°C, 5% CO_2_ for 7 days. The number of colonies was counted using an EVOS fl microscope (Thermo Fisher Scientific).

#### Histological analysis

Whole-mount *in situ* hybridization and immunohistochemistry on a paraffin section were performed as previously described.^35^ Digoxigenin (DIG)-labeled RNA probes were prepared by *in vitro* transcription with linearized constructs using DIG RNA Labeling Mix and T7 RNA polymerase (Sigma). The trunk body containing the kidney was fixed with 4% paraformaldehyde (PFA) in PBS for 2 h at room temperature. The kidney was then carefully dissected, re-fixed with 4% PFA at 4°C for overnight, and washed with 0.1% Tween-20 (Sigma) in PBS (PBST). The kidney was then treated with 6% H_2_O_2_ in PBST for 1 h to remove the melanin. Embryos were fixed in 4% PFA in PBS and washed with PBST. For permeabilization, kidneys and embryos were treated with proteinase K (10 μg/ml) (Sigma) for 30 and 4 min, respectively, refixed with 4% PFA, and washed with PBST. Hybridization was then performed using DIG-labeled antisense RNA probes diluted in hybridization buffer (50% formamide, 5X standard saline citrate (SSC), 0.1% Tween-20, 500 μg/mL torula RNA, 50 μg/mL heparin) for 3 days at 65°C. For detection of DIG-labeled RNA probes, embryos were blocked in 0.2% bovine serum albumin (BSA) (Sigma) in PBST and incubated overnight at 4°C with the alkaline phosphatase-conjugated anti-DIG antibody (Roche) at a dilution of 1:5000. After washing with PBST, embryos were developed using nitroblue tetrazolium chloride and 5-bromo-4-chloro-3-indolyl-phosphate (NBT/BCIP) (Roche) in staining buffer (100 mM Tris pH9.5, 100 mM NaCl, 50 mM MgCl_2_, 0.1% Tween-20). For fluorescent whole-mount *in situ* hybridization, kidneys were blocked in 2% blocking reagent (Roche) in 1X maleic acid buffer and incubated overnight at 4°C with the peroxidase-conjugated anti-DIG antibody (Sigma) at a dilution of 1:500. After washing with PBST, kidneys were developed using TSA Plus Cyanine 5 System (Perkin Elmer).

For whole-mount immunohistochemistry, fixed kidneys were used for fructose-based clearing method.^55^ Kidneys were immersed in a solution of 20, 40, 60, 80, and 100% (w/v) fructose in distilled water for at least 4 h at room temperature, followed by incubation in SeeDB solution, containing 20.25g fructose and 100 μL thioglycerol in 5 mL distilled water, for overnight at room temperature. Kidneys were then blocked with 2% blocking reagent in 1X maleic acid buffer for 1 h at room temperature.

For tissue section immunohistochemistry, dissected kidneys were fixed with 4% PFA in PBS, embedded in paraffin, and sectioned at 3μm in thickness. Deparaffinized tissue sections were incubated with 0.2% BSA in PBS for 30 min at room temperature. For immunostaining of sorted cells, cells were smeared with Cyto-Tek 2500 Cytocentrifuge (Sakura) and fixed with 4% PFA in PBS.

For antibody staining, the chicken anti-GFP (Aves) (for GFP and Cerulean staining) and rabbit anti-RFP (Abcam) (for DsRed and mCherry staining), goat anti-chicken IgY Alexa Fluor 488-conjugated (Abcam), donkey anti-rabbit IgG Alexa Fluor 647-conjugated (Abcam), and goat anti-mouse IgG Alexa Fluor 647-conjugated (Abcam) were used at 1:1000 dilution. The mouse anti-Jam1a antibody (2O7) was generated by Abmart and used at 1:200 dilution. Primary antibody staining was performed at 4°C overnight in both whole-mount and tissue section immunohistochemistry, and secondary antibody staining was performed at 4°C overnight in whole-mount immunohistochemistry and at room temperature for 2 h in tissue section immunohistochemistry.

For hematoxylin-eosin staining, tissue sections were deparaffinized in xylene, rehydrated through graded alcohol solutions, and stained with hematoxylin and eosin.

#### Imaging

For fluorescent imaging, kidneys were mounted in a glass bottom dish filled with 0.6% low-gelling agarose (Sigma). Fluorescent images were captured using an FV10i confocal microscope and Fluoview FV10i-SW software (ver. 2.1.1) (Olympus). Visible light imaging of embryos and adult animals were captured using an Axiozoom V16 microscope (Zeiss) with a TrueChrome II digital camera (BioTools) and TCapture software (ver. 4.3.0.602) (Tucsen Photonics) and a Nikon Coolpix B500 distal camera (Nikon), respectively. Random cells were randomly selected in the confocal image data by determining their positions by random numbers for the X, Y, and Z (*gata2a*^+^ *runx1*^+^ cells) or X and Y axis coordinates (*runx1*^+^ cells).

#### Western blotting

Western blotting was performed as previously described.^35^ Wild-type and *jam1a*^*sd43*^ embryos were lysed in lysis buffer (25mM Tris-HCl pH7.4, 1mM EDTA, 0.1mM EGTA, 150mM NaCl, 5mM MgCl_2_, 2mM Na_3_VO_4_, 20% glycerol, 0.1% Triton X-100, 1mM dithiothreitol, proteinase inhibitor). Samples were separated by a NuPAGE 4-12% Bis-Tris Gel (Novax). The gel was then transferred using Semi-Dry transfer cell (BIO-RAD) to Immobilon-P Membrane (Millipore, IPVH00010). The membrane was blocked with 1% skim milk, 0.05% Tween-20 in PBS for 30 min at room temperature, and then incubated with 1:1000 mouse anti-Jam1a antibody or 1:1000 mouse anti-GAPDH antibody (Abcam) overnight at 4°C. After washing with PBST, the membrane was incubated with 1:10000 goat anti-mouse IgG HRP-conjugated secondary antibody (Jackson ImmunoResearch) for 45 min at room temperature. After washing with PBST, chemiluminescence was performed using the SuperSignal West Pico Chemiluminescent Substrate (Thermo Fisher Scientific).

### Quantification and statistical analysis

Data were analyzed for statistical significance after at least two repeated experiments. Statistical differences between groups were determined by unpaired two-tailed Student’s *t*-test, one-way ANOVA with Dunnett’s test, or chi-square statistic. A value of *p* < 0.05 was considered to be statistically significant.

## Data Availability

All data reported in this paper will be shared by the lead contact upon request. No original code was reported in this study. Any additional information required to reanalyse the data reported in this paper is available from the lead contact upon request.
