# Supplementary material for: The sinusoidal hematopoietic niche is formed by Jam1a via Notch signaling in the zebrafish kidney
Source: iScience. 2023 Mar 27;26(4):106508. doi: 10.1016/j.isci.2023.106508 (PMC10139997; doi:10.1016/j.isci.2023.106508)
Supplement: Document S1. Figures S1–S10 and Table S1 [file mmc1.pdf]

iScience, Volume *26*

Supplemental information

The sinusoidal hematopoietic niche is formed by Jam1a via Notch signaling in the zebrafish kidney

Mao Kondo, Koki Kimura, Jingjing Kobayashi-Sun, Shiori Yamamori, Makoto Taniguchi, David Traver, and Isao Kobayashi

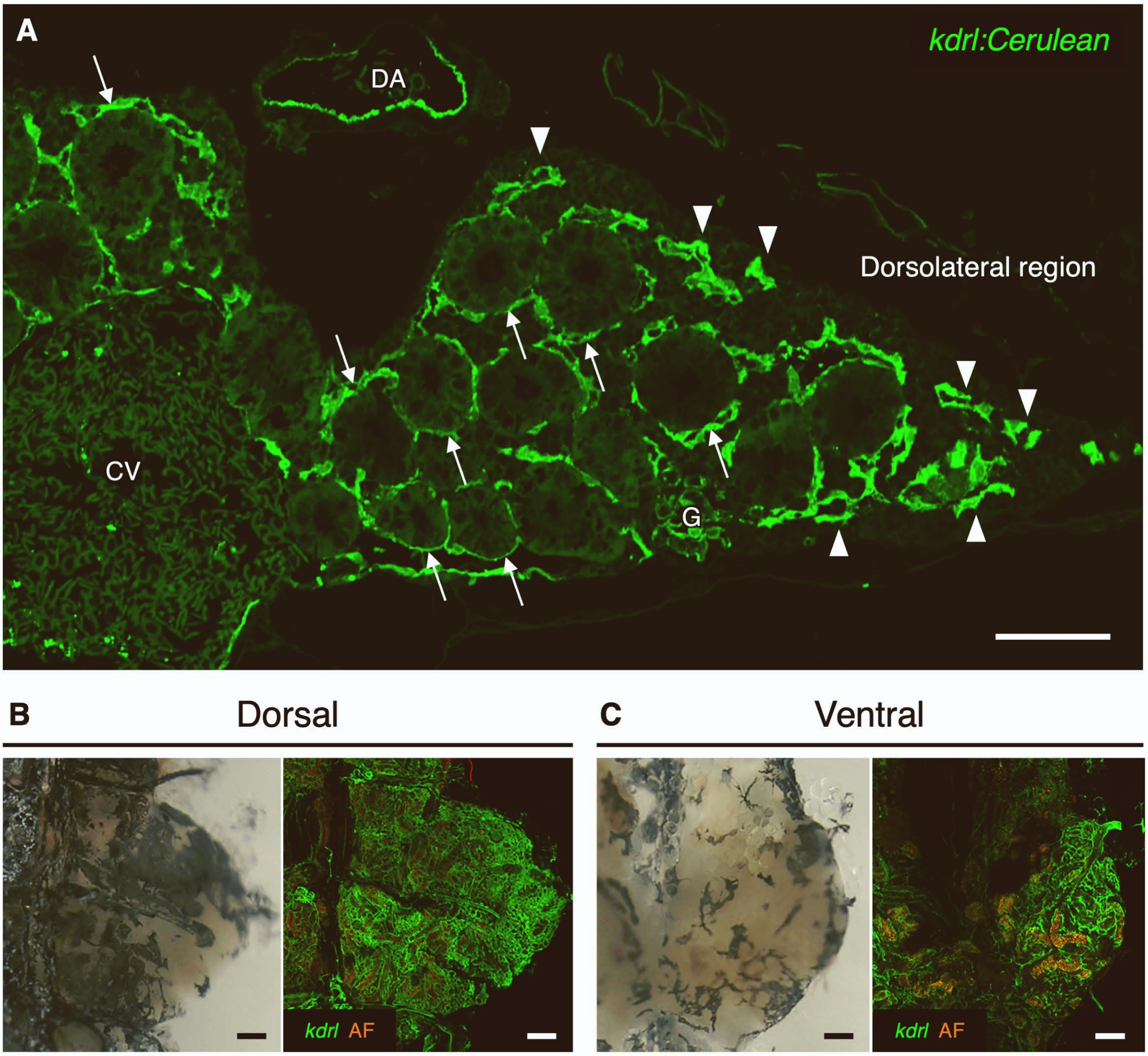

Figure S1. Sinusoids and melanocytes are predominantly observed in the dorsolateral region of the kidney (related to Figure 1 and 2)
(**A**) Transverse section of a *kdrl:Cerulean* kidney. Arrows and arrowheads denote the endothelium surrounding the renal tubule ("renal endothelium") and the sinusoidal endothelium, respectively. DA, dorsal aorta; CV, cardinal vein; G, glomerulus. (**B**, **C**) Dorsal and ventral view of a kidney under a *kdrl:Cerulean* background. Left and right panels show a brightfield image before clearing and a fluorescence image after clearing with antibody staining, respectively. AF denotes auto-fluorescence of renal tubules. Bars, 50 µm (**A**); 100 µm (**B**, **C**).

A

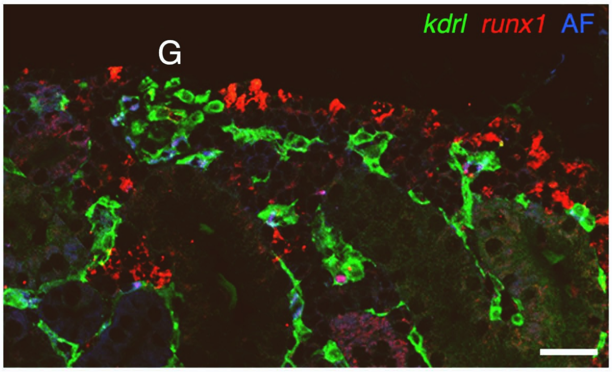

C

Glomeruli		
Region	% distribution	% within the sinusoidal region
DM	31.3	70.0
DL	31.3	90.0
VM	12.4	75.0
VL	25.0	87.5

B

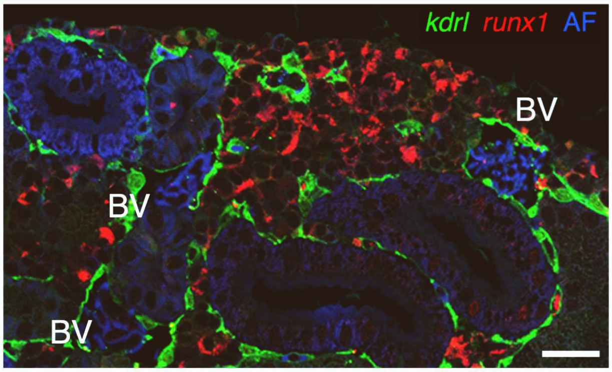

D

Blood vessels		
Region	% distribution	% within the sinusoidal region
DM	35.7	20.0
DL	42.9	83.3
VM	9.5	0.0
VL	11.9	60.0

Figure S2. Distribution of glomeruli and blood vessels in the kidney (related to Figure 1 and 2)
(**A**, **B**) Sections in the dorsolateral (DL) region of the *kdrl:Cerulean*; *runx1:mCherry* kidney. AF denotes auto-fluorescence of renal tubules or erythrocytes. G, glomerulus; BV, blood vessel. Bars, 20 µm. (**C**, **D**) Percent distribution of glomeruli or blood vessels in the DL, dorsomedial (DM), ventrolateral (VL), or ventromedial (VM) region of the kidney. Percent within the sinusoidal region indicates the percentage of glomeruli or blood vessels that were distributed within the sinusoidal region among those observed in DL, DM, VL, or VM. Percentages were calculated from 4 zebrafish.

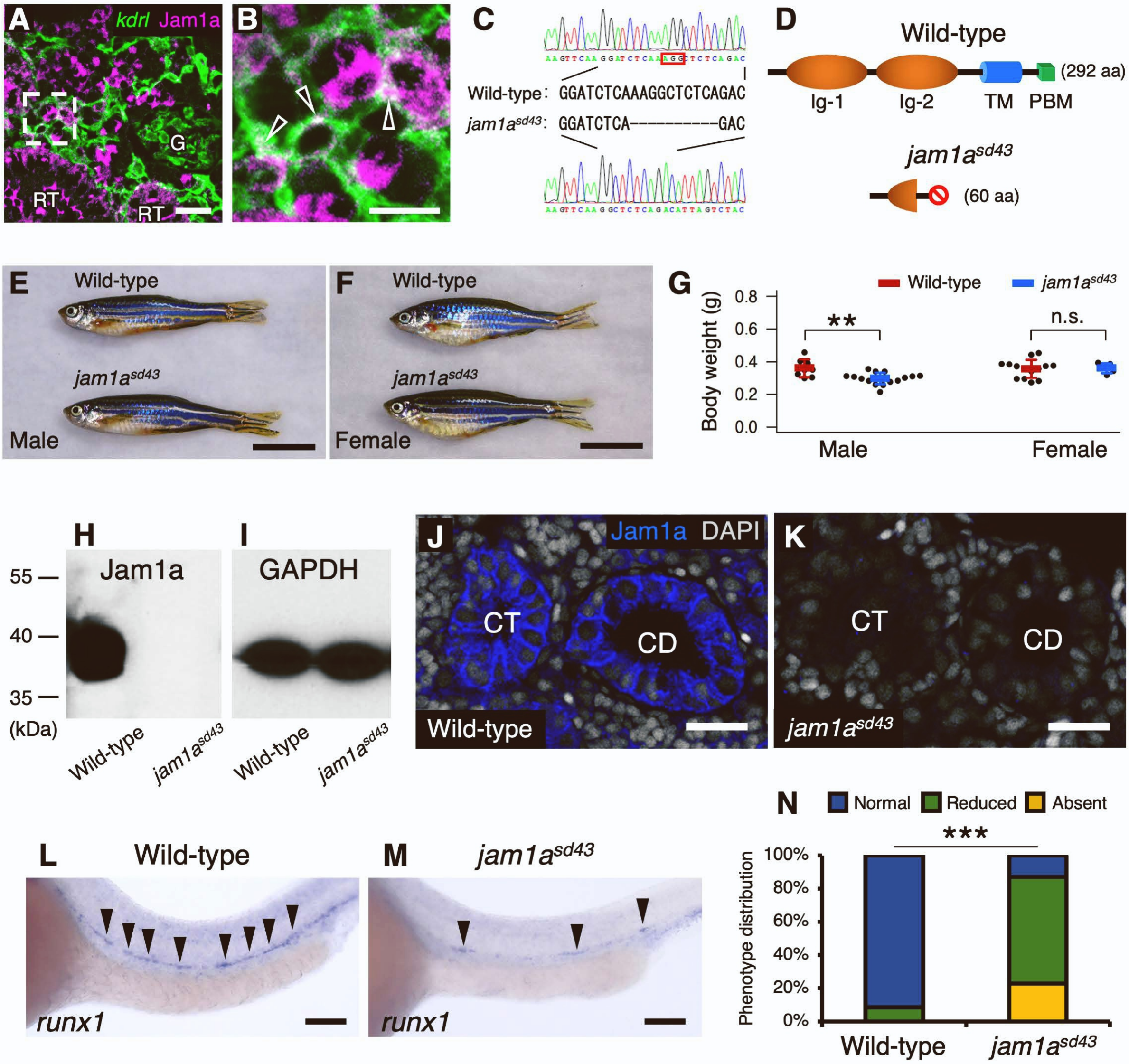

Figure S3. Characterization of *jam1a*^{sd43} zebrafish (related to Figure 3, 4, 5, and 6)
(**A**, **B**) Expression of Jam1a in kidney under a *kdrl:NTR-cerulean* background. A high magnification view of the white dotted area is shown in **B**. Arrowheads indicate Jam1a expression detected in the sinusoidal endothelium. (**C**) Mutation in exon 3 of *jam1a* was verified by sequencing in wild-type or *jam1a*^{sd43} embryos. (**D**) Schematic diagram of the predicted Jam1a protein in wild-type or *jam1a*^{sd43} animals. Ig, immunoglobulin-like domain; TM, transmembrane domain; PBM, PDZ-binding motif; aa, amino acid. (**E**, **F**) Images of wild-type and *jam1a*^{sd43} zebrafish at 4 months of age. (**G**) Body weights of wild-type and *jam1a*^{sd43} animals at 3 to 6 months of age. Error bar, s.d. (n = 8 (wild-type male), 16 (*jam1a*^{sd43} female), 14 (wild-type female), or 5 (*jam1a*^{sd43} female). (**H**, **I**) Expression of Jam1a and GAPDH in wild-type or *jam1a*^{sd43} embryos. (**J**, **K**) Expression of Jam1a in the collecting duct (CD) and tubule (CT) of wild-type or *jam1a*^{sd43} animals. (**L**, **M**) Expression of *runx1* in wild-type or *jam1a*^{sd43} embryos. Arrowheads indicate *runx1* expression in the dorsal aorta. (**N**) Quantitative phenotype distribution of *runx1* expression in wild-type (n = 36) or *jam1a*^{sd43} embryos (n = 31). Bars, 10 mm (**E**, **F**); 100 µm (**L**, **M**); 20 µm (**A**, **J**, **K**); 10 µm (**B**). $**p < 0.01$; $***p < 0.001$; n.s., no significance.

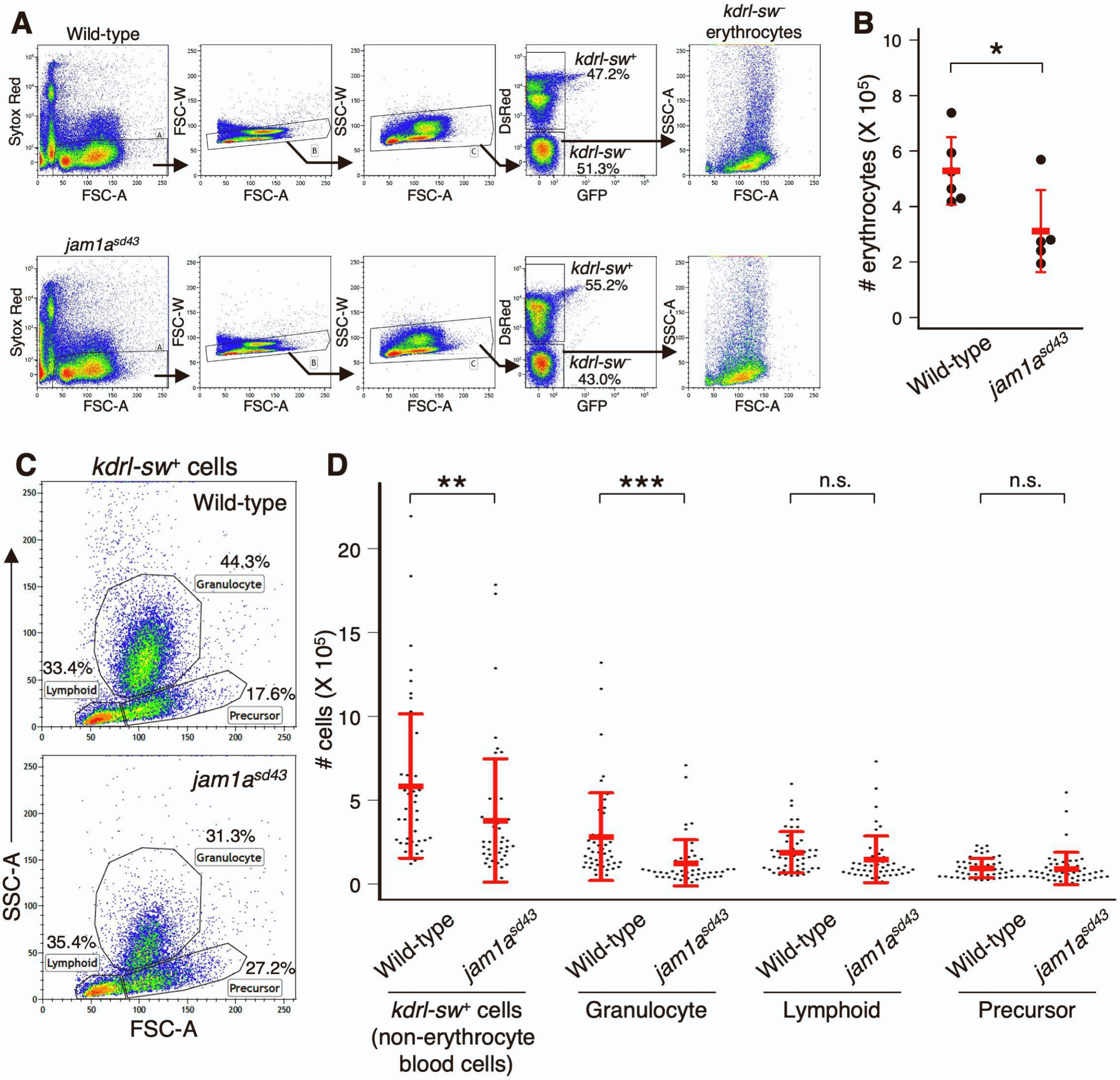

Figure S4. Blood cells are reduced in the *jam1a^{sd43}* kidney (related to Figure 3, 4, 5, and 6)

(**A**) Representative result of FCM analysis in blood cells from the wild-type or *jam1a^{sd43}* kidney under a *kdrl-sw* background. *kdrl-sw*$^{-}$ erythrocytes were displayed in an FSC vs. SSC plot. (**B**) Absolute numbers of erythrocytes in the wild-type or *jam1a^{sd43}* kidney (mean ± s.d.; n = 6 (wild-type) or 5 (*jam1a^{sd43}*)) (**C**) *kdrl-sw*$^{+}$ cells (non-erythrocyte blood cells) were displayed in an FSC vs. SSC plot to resolve three distinct populations, "granulocyte", "precursor", and "lymphoid". (**D**) Absolute numbers of cells in each fraction of the wild-type or *jam1a^{sd43}* kidney (mean ± s.d.; n = 53 (wild-type) or 52 (*jam1a^{sd43}*)). n.s., no significance; *$p < 0.05$; **$p < 0.01$; ***$p < 0.001$.

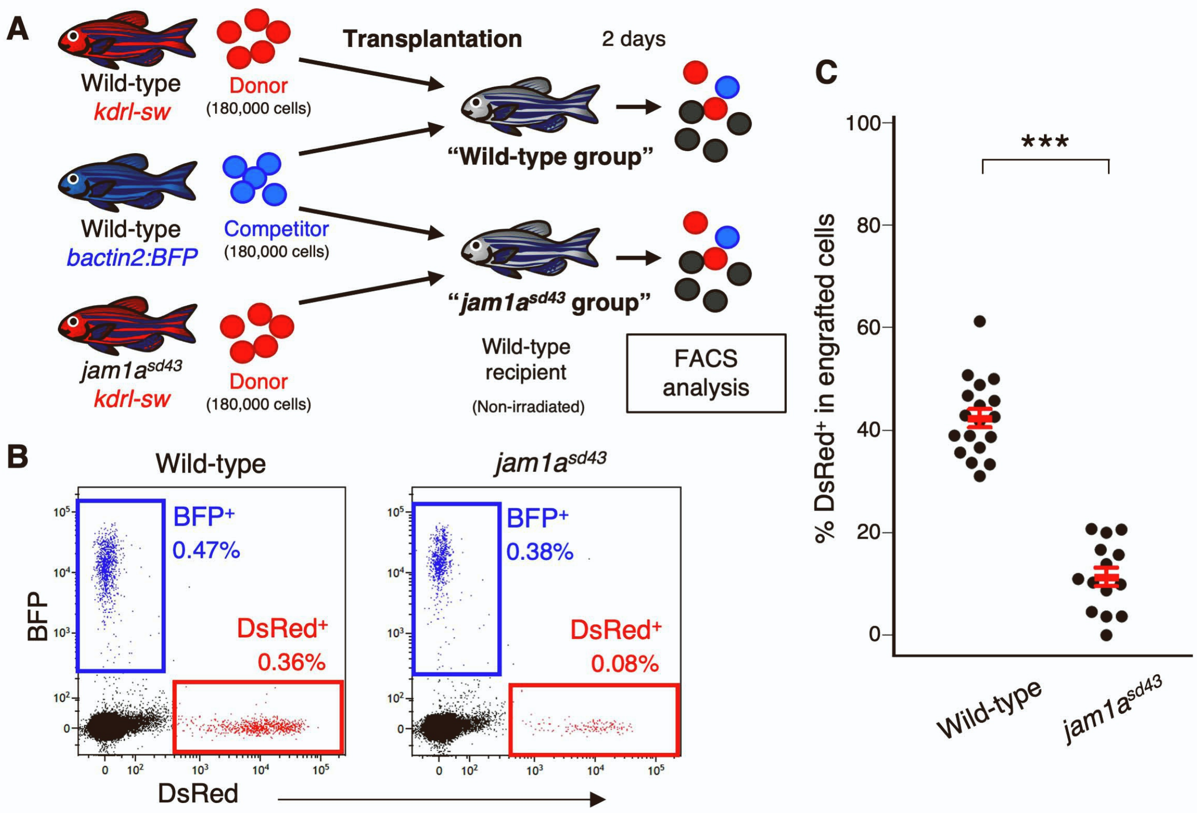

Figure S5. Homing assay for wild-type and *jam1a^sd4^* KMCs (related to Figure 3, 4, 5, and 6)

(**A**) Experimental procedure for homing assay. DsRed-labeled KMCs from wild-type or *jam1a*^{sd43} animals were co-transplanted with equivalent numbers of BFP-labeled wild-type KMCs into wild-type recipients (wild-type or *jam1a*^{sd43} group). At 2 days post-transplantation, KMCs from each recipient group were analyzed by FCM. (**B**) Representative result of FCM analysis in recipients of wild-type or *jam1a*^{sd43} group. (**C**) Percentages of DsRed^{+} cells within the total BFP^{+} or DsRed^{+} cells in each group (mean ± s.e.m; n = 18 (wild-type) or 14 (*jam1a*^{sd43})). ***$p < 0.001$.

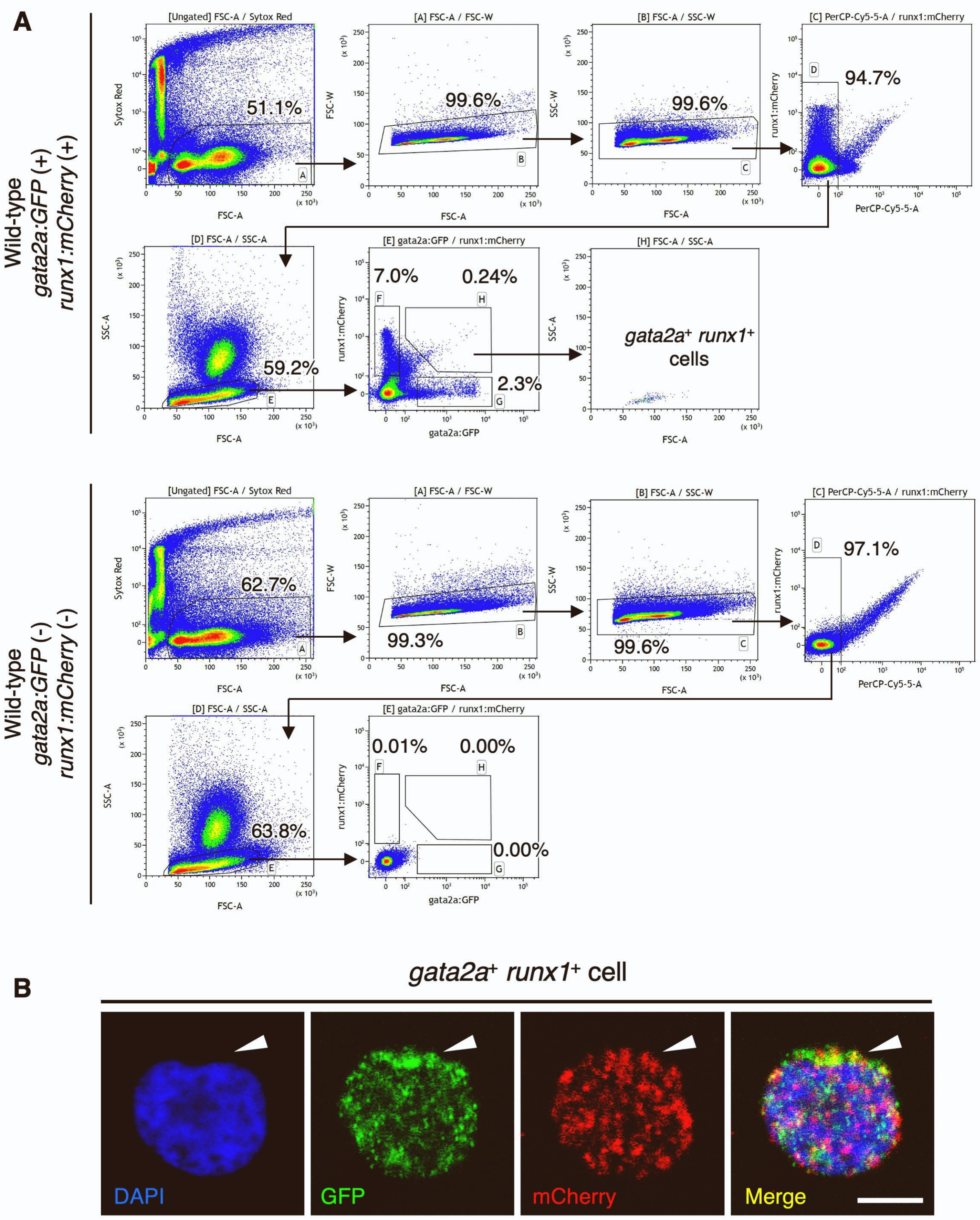

Figure S6. Gating strategy and morphology of *gata2a*⁺ *runx1*⁺ cells (related to Figure 4) (**A**) Representative result of FCM analysis in KMCs from wild-type animals under *gata2a:GFP* (+); *runx1:mCherry* (+) or *gata2a:GFP* (–) *runx1:mCherry* (–) background. The PerCP-Cy5-5 filter was used to exclude cells with autofluorescence. (**B**) Immunostaining of a *gata2a*⁺ *runx1*⁺ cell using anti-GFP and anti-mCherry antibodies. Expression of both GFP and mCherry was detected throughout the cell, including in the cytoplasm (arrowheads). Bar, 5 µm.

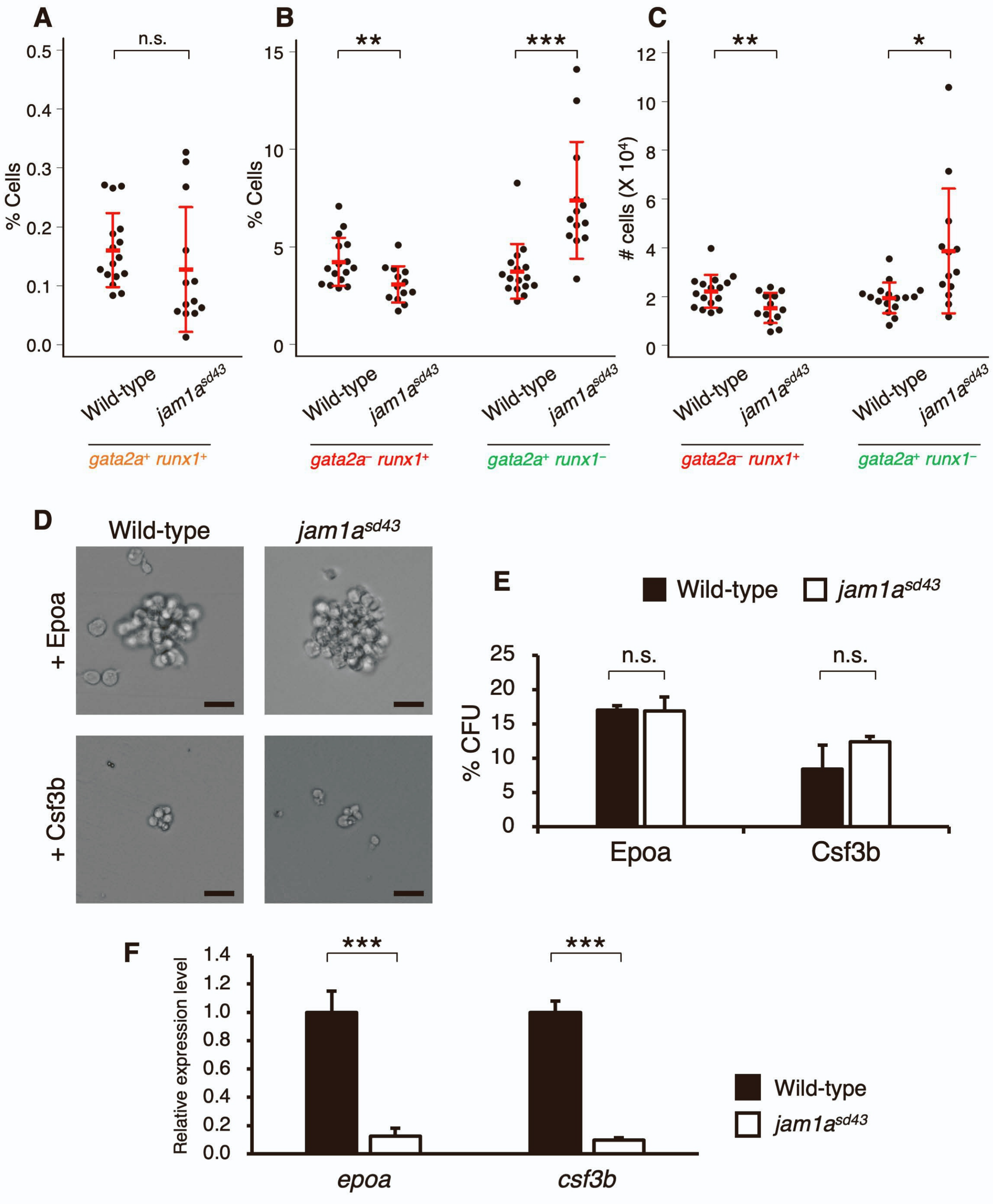

Figure S7. Differentiation potential of *gata2a*$^{+}$ *runx1*$^{+}$ cells is unaffected in *jam1a*^{sd43} animals (related to Figure 3, 4, 5, and 6)

(**A-C**) Percentages and/or absolute numbers of *gata2a*$^{+}$ *runx1*$^{+}$, *gata2a*$^{-}$ *runx1*$^{+}$, and *gata2a*$^{+}$ *runx1*$^{-}$ cells in the wild-type or *jam1a*^{sd43} kidney (mean ± s.d. (n = 16 (wild-type) or 13 (*jam1a*^{sd43})). (**D**, **E**) Colony assay of *gata2a*$^{+}$ *runx1*$^{+}$ cells of wild-type or *jam1a*^{sd43} animals. Representative images of a colony (**D**) and percentages of CFU in *gata2a*$^{+}$ *runx1*$^{+}$ cells of wild-type or *jam1a*^{sd43} animals in the presence of Epoa or Csf3b (**E**) are shown (mean ± s.d.; n = 3 for each). Bars, 20 µm. (**F**) Relative expression of *epoa* and *csf3b* in the wild-type or *jam1a*^{sd43} kidney. Error bars, s.d. (n = 4 for each). *$p < 0.05$; **$p < 0.01$; ***$p < 0.001$.

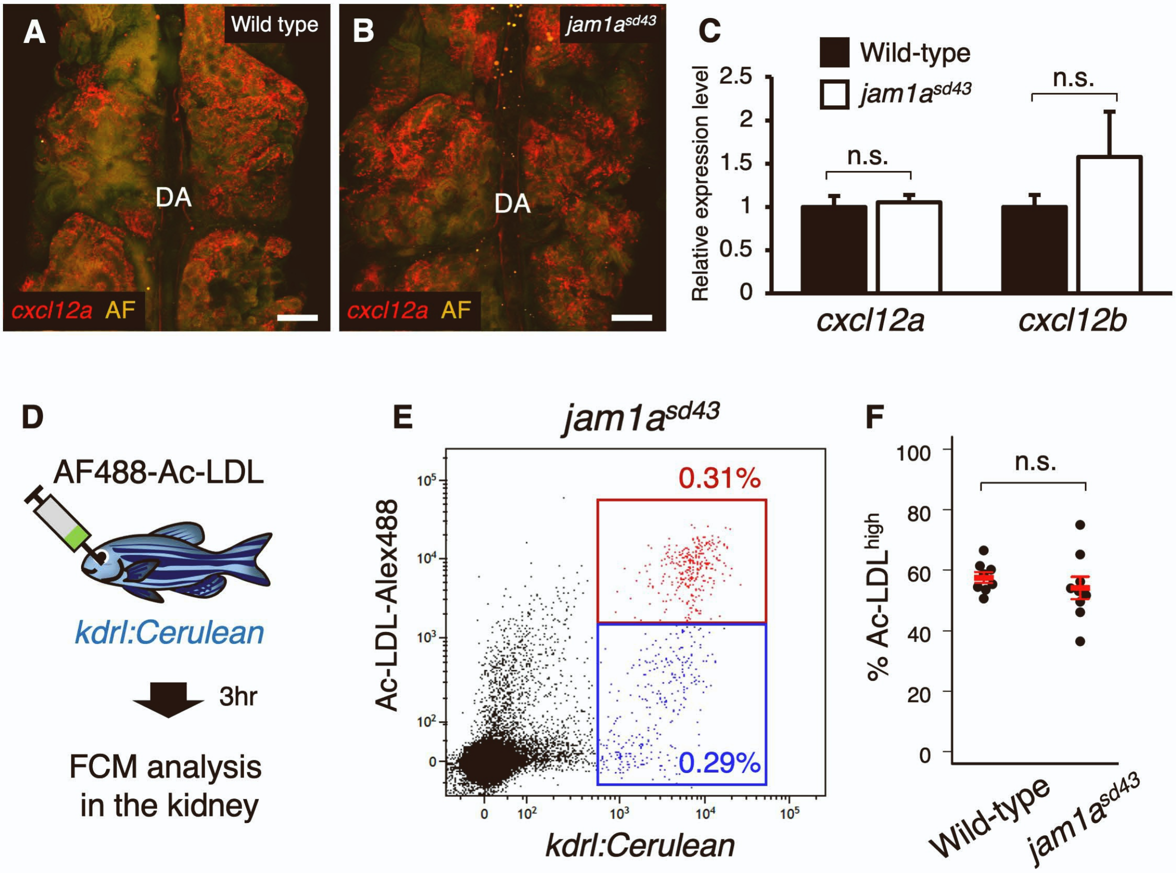

Figure S8. *cxcl12* expression and percentage of Ac-LDL^{high} cells in the *jam1a^{sd43}* kidney (related to Figure 6)

(**A**, **B**) Expression of *cxcl12a* in a wild-type or *jam1a^{sd43}* kidney. AF denotes auto-fluorescence of renal tubules. DA, dorsal aorta. Bars, 200 μm. (**C**) Relative expression of *cxcl12a* and *cxcl12b* in the wild-type or *jam1a^{sd43}* kidney. Error bars, s.d. (n = 4 for each). (**D**) Experimental procedure for isolation of sinusoidal endothelial cells in kidneys. (**E**) Representative result of FCM analysis in *jam1a^{sd43} kdrl:Cerulean* animals injected with AF488-Ac-LDL. (**F**) Percentages of the Ac-LDL^{high} fraction within *kdrl:Cerulean*$^{+}$ cells in wild-type or *jam1a^{sd43}* kidneys. Error bars, s.e.m. (n = 8 (wild-type) or 9 (*jam1a^{sd43}*)). n.s., no significance.

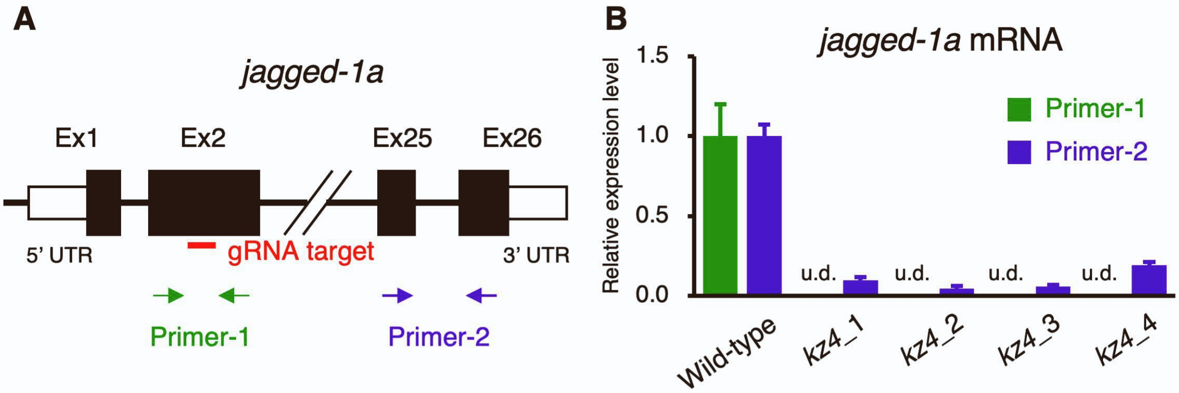

Figure S9. Generation of *jagged-1a* mutant zebrafish (related to Figure 7)
(**A**) Schematic diagram of the genomic loci of *jagged-1a*. The gRNA target was designed in Exon 2, and two sets of qPCR primers were designed in Exon 2 and Exon 25-26. (**B**) Expression of *jagged-1a* in individual wild-type or *jagged-1a*^{kz4} kidneys (*kz4*_1 – 4). Error bars, s.d. (n = 4 for each). u.d., undetected.

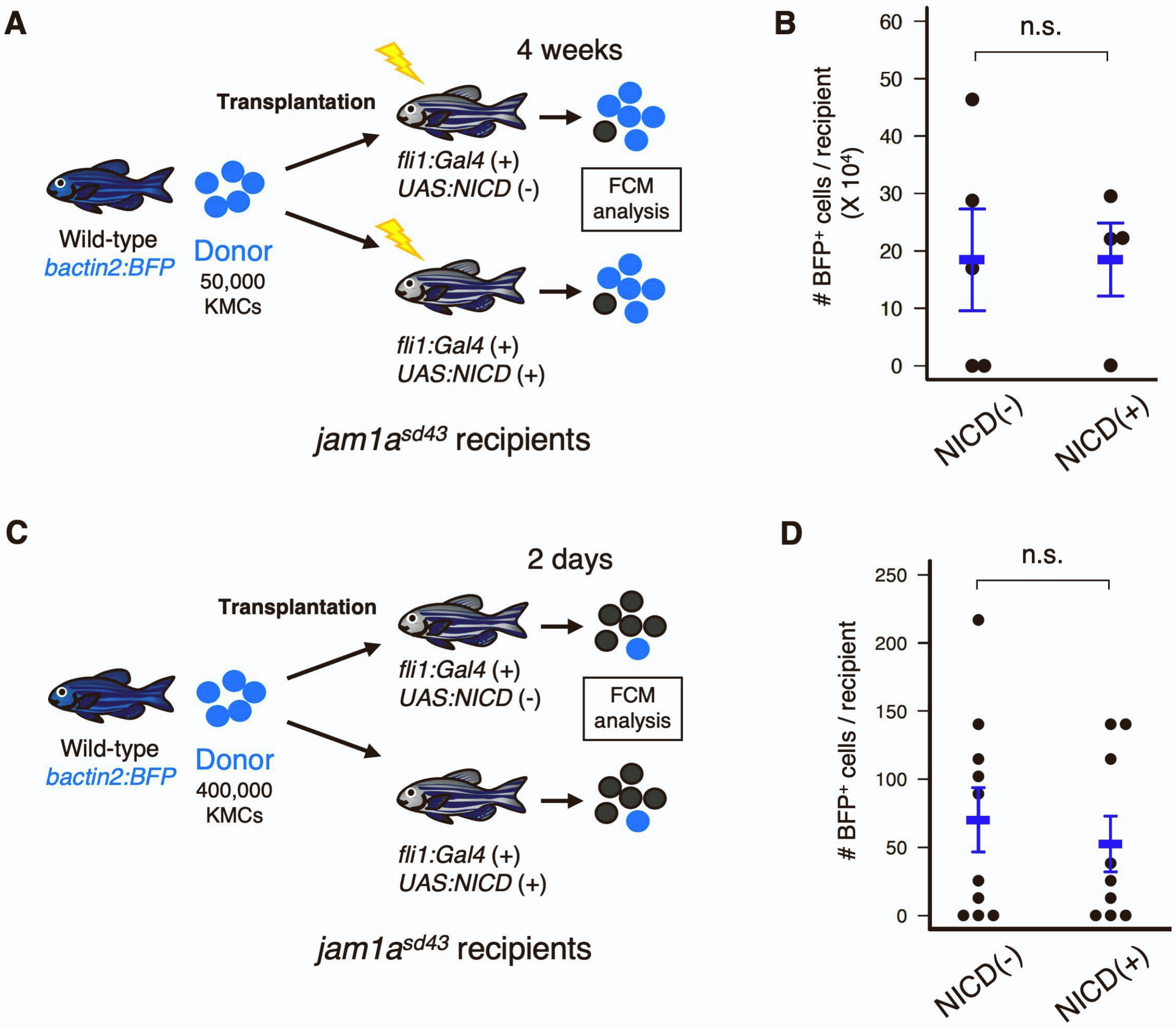

Figure S10. Forced expression of NICD did not recover niche functions in the *jam1a*^{sd43} kidney (related to Figure 8)

(**A**) Experimental procedure for transplantation assay using *jam1a*^{sd43} recipients expressing or not NICD. (**B**) Absolute numbers of BFP^{+} cells in *jam1a*^{sd43} recipients expressing or not NICD (mean $\pm$ s.e.m; n = 5 (NICD (-)) or 4 (NICD (+))). (**C**) Experimental procedure for homing assay using *jam1a*^{sd43} recipients expressing or not NICD. (**D**) Absolute numbers of BFP^{+} cells in *jam1a*^{sd43} recipients expressing or not NICD (mean $\pm$ s.e.m; n = 10 (NICD (-)) or 9 (NICD (+))). n.s., no significance.

Table S1. Primer and oligo sequences (related to STAR Methods)

Gene	gRNA target sequence	Description
jam1a	GGAAGTTCAAGGATCTCAA	gRNA
jagged-1a	GCGGAGCGCTCCTGCGCGGC	gRNA

Gene	Forward primer	Reverse primer	Description
jam1a	TTCATTTTGGACGCATCCTT	TGGCTTGCCATCAAAGTAGA	Genotyping of *jam1a*^{sd43}
jagged-1a	AGAATGTGAACGGGGAACTG	CAAGGGGCTTGACAGAAAAA	Genotyping of *jagged-1a*^{kz4}
jagged-1a	ACGGAAGCGGATCTACTCCT	GTGTTTCAGGACCTGCCATT	qPCR
cxcl12a	CCCATCAGCCTGGTAGAGAG	GTTGATGGCGTTCTTCAGGT	qPCR
cxcl12b	CTTCTGGAGCCCAGAGACTG	TGTTCTTCAGCTTGGCAATG	qPCR
kitlga	TCCCACATCCACAAAAACAA	GGTGAGGAGCCACATGAGAT	qPCR
angpt1	GGAAACAGCACAGCACTTCA	TTTCTGGAAGCCTGCTTGAT	qPCR
jagged-1b	TGGTGAGCAAGCATAATGGA	GTGTTGCTGTGGGTGTTTTG	qPCR
vcam1b	AGCAAGCTGGATAGCGAGAC	AAGCAGAGCAGCAGAACCTC	qPCR
sele	GGCAGCTCCAGTCTGTTCTT	CATGGAAAATGCCCATCATA	qPCR
cx43	GTCGCGTACTTGGATTTGGT	CACAGAGAGCCAGACCTTCC	qPCR
her2	CGAAATAAAGGCCAGTCAGC	CCGTTTCCTCAATGCATCTT	qPCR
her6	GGTTAACACCGAGGTCAGGA	TGAACCATGGGTTGACTGAA	qPCR
hey1	TGTGCATATGTTTGCCCTTT	AGGGACAGGCACAGTACCAC	qPCR
hey2	CAAGTTGGAGAAAGCGGAAA	CTGGCCACTTCAGTCAGACA	qPCR
epoa	TACTGCTGATGGTGCTGGAG	TGGTCAGAGGAACAGTGACG	qPCR
csf3b	CCTTGAGTTTGGCCAAGAAG	AGCAGCTTCAGCAGGACTCT	qPCR
ef1a	ACCGGCCATCTGATCTACAA	CAATGGTGATACCACGCTCA	qPCR

Primer sequence for whole-transcript amplification	Description
TATAGAATTCGCGGCCGCTCGCGATAATACGACTCACTATAGGGCGTTTTTTTTTTTTTTTTTTTTTTTT	RT primer
TATAGAATTCGCGGCCGCTCGCGATTTTTTTTTTTTTTTTTTTTTTTTT	Tagging primer
(5' Aminolink)-GTATAGAATTCGCGGCCGCTCGCGAT	Suppression primer
